# Risk of bias in machine learning and statistical models to predict height or weight: a systematic review in fetal and paediatric medicine

**DOI:** 10.1186/s41512-025-00215-6

**Published:** 2025-12-15

**Authors:** Neil R Lawrence, Irina Bacila, Joseph Tonge, Anthea Tucker, Jeremy Dawson, Zi-Qiang Lang, Nils P Krone, Paula Dhiman, Gary S Collins

**Affiliations:** 1 Division of Clinical Medicine, University of Sheffield, Sheffield, S10 2RX UK; 2 Division of Medicine, Sheffield Children’s Hospital, Sheffield, S10 2TH UK; 3 Health Sciences Library, University of Sheffield, Sheffield, S10 2RX UK; 4 Division of Population Health, University of Sheffield, Sheffield, S10 2RX UK; 5 Management School, University of Sheffield, Sheffield, S10 2RX UK; 6 Department of Automatic Control and Systems Engineering, University of Sheffield, Sheffield, S1 3JD UK; 7 Centre for Statistics in Medicine, Nuffield Department of Orthopaedics, Rheumatology and Musculoskeletal Sciences, University of Oxford, Oxford, OX3 7LD UK; 8 Department of Applied Health Sciences, University of Birmingham, Birmingham, B15 2TT United Kingdom

**Keywords:** Risk of bias, Systematic review, Prediction modelling, Statistical modelling, Machine learning, Height, Weight, Growth, Obstetrics, Paediatrics

## Abstract

**Background:**

Prediction of suboptimal growth allows early intervention that can improve outcomes for developing fetus’ as well as infants and children. We investigate the risk of bias in statistical or machine learning models to predict the height or weight of a fetus, infant or child under 20 years of age to inform the current standard of research and provide insight into why equations developed over 30 years ago are still recommended for use by national professional bodies.

**Methods:**

We systematically searched MEDLINE and EMBASE for peer reviewed original research studies published in 2022. We included studies if they developed or validated a multivariable model to predict height or weight of an individual using two or more variables, excluding studies assessing imaging or using genetics or metabolomics information. Risk of bias was assessed for all prediction models and analyses using the Prediction model Risk Of Bias ASsessment Tool (PROBAST).

**Results:**

Sixty-four studies were included, in which we assessed the development of 180 models and validation of 61 models. Sample size was only considered in 10% of developed models and 13% of validated models. Despite height and weight being continuous variables, 77% of models developed predicted a dichotomised outcome variable.

**Registration:**

The review was registered on PROSPERO (ID: CRD42023421146), the International prospective register of systematic reviews on 26/4/2023.

**Supplementary Information:**

The online version contains supplementary material available at 10.1186/s41512-025-00215-6.

## Background

Impaired growth in the early years of life is reflective of poor health, and a predictor of poor long term outcomes [1–3]. Identifying pregnancies where the fetus has excessive growth can improve birth outcomes by controlling maternal comorbidities and guiding decisions about caesarean section [4]. Monitoring of postnatal and childhood growth is equally important, with adverse growth associated with pathology, malnutrition, and neglect [5, 6].

Prediction of suboptimal growth allows for early intervention that can improve prognosis [7]. The application of ultrasound revolutionised antenatal birthweight prediction [8], the Hadlock formulae developed in the 1980s facilitating accurate prediction that is still widely used today, recommended in the UK, Canada and United States by their respective national obstetric advice bodies [2, 9–12]. Statistical tables to predict growth using bone age in children by Bayley and Pinneau were first published in 1946 [13], and seminal work was conducted by Tanner et al. to inform predictions using parental height in the 1970s [14, 15]. Adult height prediction in children recommended by the UK Royal College of Paediatrics and Child Health is via a prediction model developed in 2011 [16], although the American Academy of Paediatrics recommend employing x-ray quantification of bone age, one of the most widely used being a model first developed in 2009 [17].

The development of prediction models in medicine is increasing exponentially, with a growing proportion employing machine learning methods [18, 19], despite often not demonstrating additional predictive performance over more transparent techniques such as logistic regression [20]. Only a fraction of prediction models undergo external validation, and even fewer translate into clinical practice [21]. Health inequalities can be perpetuated if prediction models are applied in groups of individuals that are poorly represented in development data, regardless of methodology [22]. Understanding the limitations of traditional statistical techniques and machine learning helps ensure appropriate application.

The Prediction model Risk Of Bias ASsessment Tool (PROBAST) has been developed to help assess risk of bias in the development or validation of prediction models [23, 24]. To date, there has been no systematic assessment of novel algorithms to predict growth in fetuses or children. We investigate the risk of bias in statistical or machine learning models recently published to predict height or weight of a fetus, infant or child under 20 years of age to provide insight into why some equations developed over 30 years ago are still recommended for use by national professional bodies [25–27]. 

## Methods

We systematically reviewed the literature by searching MEDLINE and EMBASE using the OVID library interface on 27/4/2023. This report follows the transparent reporting of multivariable prediction models for individual prognosis or diagnosis checklist for systematic reviews and meta-analyses (TRIPOD-SRMA) (Supplementary checklist) [28]. The review was registered on PROSPERO (ID: CRD42023421146), the International prospective register of systematic reviews on 26/4/2023 [29].

### Search strategy

Search terms were constructed with the help of an information specialist (AT) using a strategy that combined four groups of terms, all of which would be included in eligible studies, combined with the Boolean operator ‘OR’. The four groups included statistical modelling or machine learning methods, model performance terms, terms relating to height or weight, and terms relating to the age of participants. These groups were combined with the Boolean operator ‘AND’, and restricted to articles published in 2022 to ensure a contemporary sample were assessed. The full search strategy is detailed in Table S1.

### Eligibility criteria

We included peer reviewed original research studies published in English that reported the development or validation of a model to predict height or weight of an individual, restricted to those published in 2022 to provide a contemporary reflection of the studies that are developing and validating these models. Studies were included if models used two or more predictor variables, with continuous or categorical outcomes, using data from any study design. We use the term ‘model’ to describe any statistical model, equation or machine learning algorithm that produces a predicted outcome designed to apply to an individual.

Included models could employ metrics derived from imaging (e.g. ultrasound), but models predicting results from images were excluded to avoid quantitative medical imaging interpretation studies. Models using composite outcomes that included height or weight alongside other outcomes were excluded to ensure homogenous comparisons. Models incorporating genomics or metabolomics were excluded to ensure models assessed used predictors that were accessible to clinicians. Studies where the focus was to identify predictors, rather than develop a model to predict an outcome in an individual, were excluded, alongside reviews and conference abstracts to evaluate models at a stage of development suitable for potential application in clinical practice.

### Selection process

Titles and abstracts were screened independently by two authors (NRL and JT), with full articles read and models assessed independently by two authors (NRL and IB). Disagreements were arbitrated by a third author (PD).

### Risk of bias assessment

PROBAST was used to assess each prediction model in four domains, each consisting signalling questions answered as ‘yes’, ‘probably yes’, ‘no information’, ‘probably no’ or ‘no’. Questions prompt consideration of risks of bias within each domain, but need not be exclusively answered positively or negatively to define the domain rating, which may be ‘low’, ‘unclear’, or ‘high’. If any domain is rated ‘high’, overall assessment of the model is high risk of bias (Tables S2-S3 outline PROBAST criteria). Model ratings were discussed between both authors to reach agreement on each screening question and domain rating, any disagreements arbitrated by a third author (PD).

### Data collection and analysis

A data collection form was developed using Excel (Microsoft, Redmond, Washington) (https://tinyurl.com/3aazxyen). We collected whether the studied population was maternal or paediatric, the outcome variable(s) of interest, geographical origin of the data, number of eligible models reported and the following model specific data items:

For each eligible model:

- Type of prediction model study (development or validation).
- Whether sample size was considered.
- Number of participants used in analysis, with number of ‘events’ for categorical outcomes.
- Whether analysis assessed discrimination, calibration, or both.
- PROBAST signalling question answers and risk of bias rating.

For models developed:

- Number of candidate predictor parameters.
- Modelling methodology employed.
- Whether final model equation or equivalent that would allow external validation of the model was reported.

Summary statistics were calculated using *R: A language and environment for statistical computing* (https://www.R-project.org), and reported with lower quartile, median and upper quartile values. Percentages were calculated for the number of models rated in each category. Models were subdivided into type of methodology (regression-based methods, flexible machine learning methods and ensemble machine learning methods) to assess for any difference in the risk of bias between categories.

## Results

### Number of studies and models assessed

**Fig. 1:**
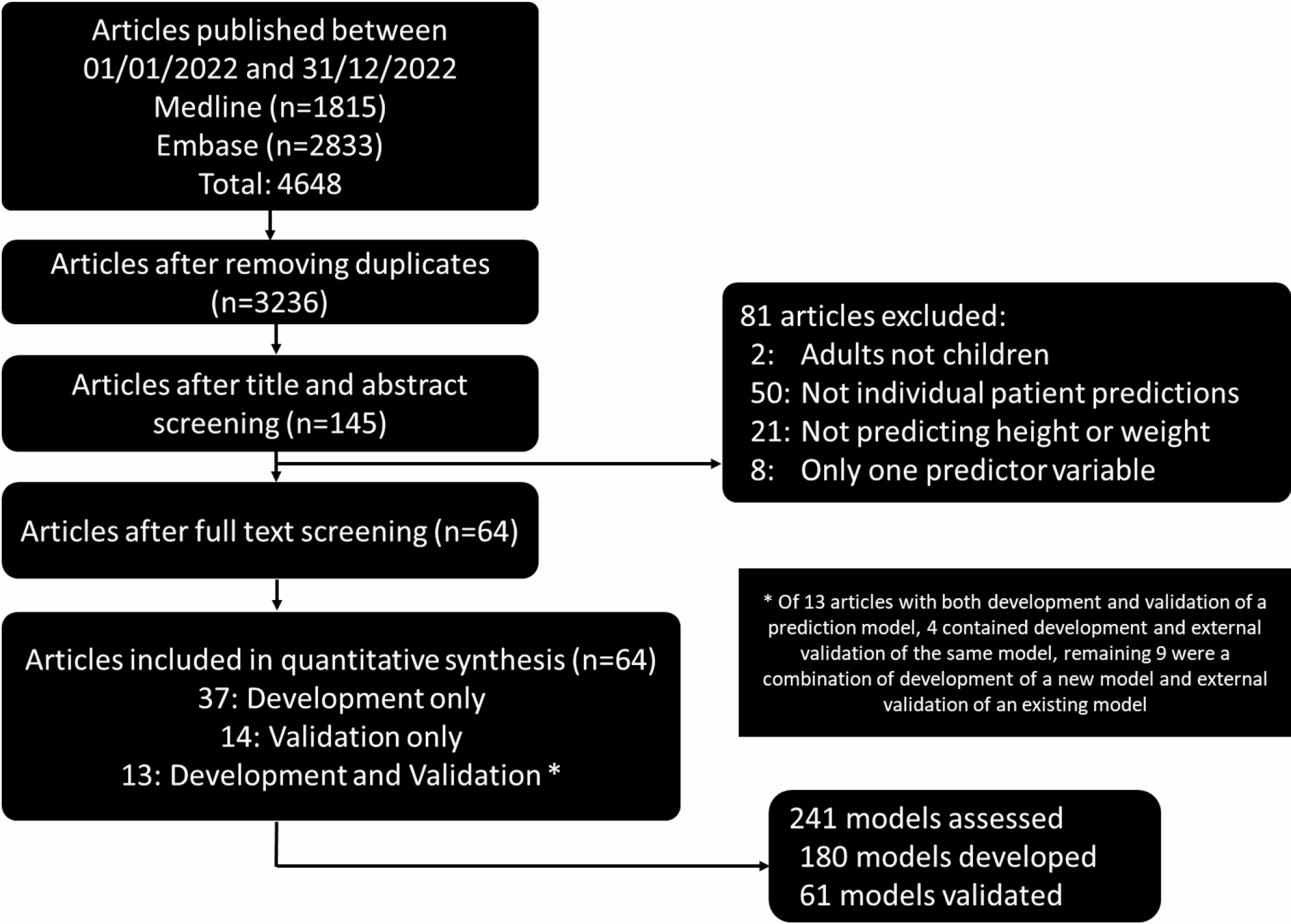
PRISMA flow diagram of included studies

The search returned 3236 eligible articles after deduplication (Fig. 1), with abstract screening returning 145 articles. At least one model that satisfied inclusion criteria was found in 64 articles (Table S4). Data used to develop and validate the models originated from participants in 26 different countries, most commonly China (*n* = 20/64, 31%) (Table 1, Table S5, Figure S1). Model development was the focus of 37 articles (58%), model validation in 14 articles (22%), and 13 featured both development and validation suitable for rating (20%). A median of 3 models were rated in each article (Q1 to Q3: 1 to 4.5, range: 1 to 25). In all articles with multiple models, the same dataset was used either with different predictors, a different outcome variable, a subset of the sample or a different methodology.

**Table 1:** Characteristics of studies included in review

	Studies including model development only *n* = 37	Studies including model validation only *n* = 14	Studies including both model development and validation *n* = 13	All studies *n* = 64
Population studied	Number of studies (%)			
Maternal population	20 (54.1%)	11 (78.6%)	8 (61.5%)	39 (60.9%)
Individuals under 18 years	17 (45.9%)	3 (21.4%)	5 (38.5%)	25 (39.1%)
Outcome of interest				
Height	9 (24.3%)	2 (14.3%)	3 (23.1%)	14 (21.9%)
Weight	23 (62.2%)	11 (78.6%)	8 (61.5%)	42 (65.6%)
BMI	4 (10.8%)	0 (0.0%)	1 (7.7%)	5 (7.8%)
Multiple outcomes^†^	1 (2.7%)	1 (7.1%)	1 (7.7%)	3 (4.7%)
**Geographical location**				
Africa	2 (5.4%)	1 (7.2%)	0 (0.0%)	3 (4.7%)
Asia	24 (64.9%)	5 (35.7%)	5 (38.4%)	34 (53.1%)
Australia	1 (2.7%)	0 (0.0%)	1 (7.7%)	2 (3.1%)
Europe	6 (16.2%)	4 (28.5%)	4 (30.8%)	14 (21.9%)
North America	3 (8.1%)	3 (21.4%)	3 (23.1%)	9 (14.0%)
Multiple: North America and Europe	1 (2.7%)	0 (0.0%)	0 (0.0%)	1 (1.6%)
Multiple: South America, North America, Europe and Asia	0 (0.0%)	1 (7.2%)	0 (0.0%)	1 (1.6%)

^†^Studies investigating multiple outcomes included height and weight in childhood in one article; height, weight, and BMI in childhood in one article; weight and length in infancy in 1 article

### Methods employed

The final model equation or code that would enable external validation of the model was provided for only 53/180 (29%) of the developed models. Logistic regression was most commonly used to develop models (*n* = 63/180, 35%), multiple linear regression second most common (23/180, 13%), with support vector machines and random forest used next most frequently (16/180, 9% each) (Figure S2). Models were categorised as regression-based methods (97/180, 53%), flexible machine learning methods (45/180, 25%) and ensemble methods (35/180, 20%), remaining models (3/180, 2%) developed with unclear methodology (Table S6). Both discrimination and calibration were assessed in 28/180 (16%) models developed, and 10/61 (16%) models validated with 122/180 (68%) models developed and 32/61 (52%) models validated relying on discrimination alone (Figure S3).

### Sample size

Sample size was only considered in 10% of developed models and 13% of validated models. Sample size methodology ranged from expert opinion to cited literature but was never consistent with the most up to date guidance [30–34]. A median of 1993 participants (Q1 to Q3: 286 to 6363) were used for model development. A continuous outcome was predicted in 42/180, the remaining 138/180 predicting a binary outcome, of which the number of outcome events used to develop 49/138 models was unclear. Of the 89/138 binary outcome models developed that clearly reported the number of outcome events, a median of 130 participants (Q1 to Q3: 78 to 412) had the outcome of interest. Where it could be calculated (84/138), there were a median of 11.7 events per candidate variable (Q1 to Q3: 4.1 to 24.7) for models predicting a binary outcome.

The sample size used to validate 5/61 models was unclear, with 56/61 using data from a median sample size of 374 (Q1 to Q3: 170 to 660). A continuous outcome variable was predicted in 30/61 models. For models predicting a binary outcome, the sample size contained a median of 85 (Q1 to Q3: 68 to 147) events.

### Risk of bias ratings

#### Overall PROBAST ratings

**Fig. 2:**
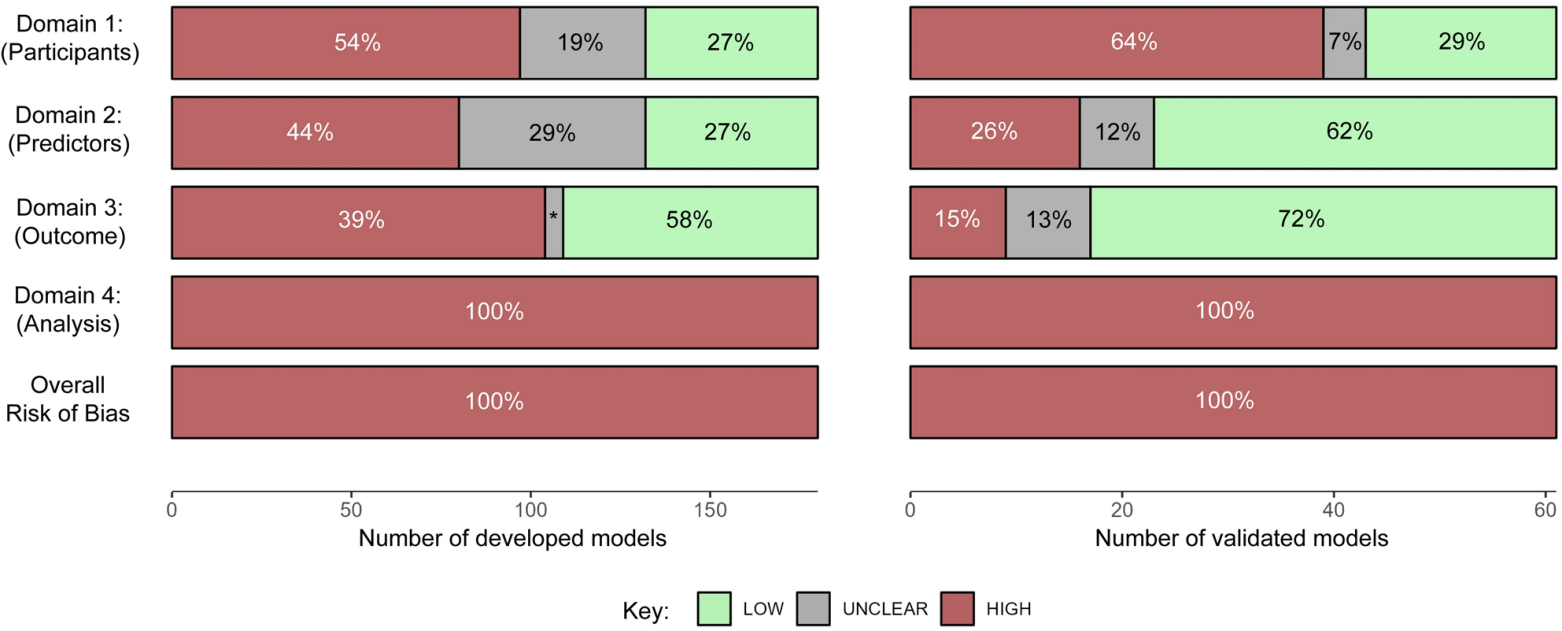
Bar chart showing risk of bias ratings by specific domain and overall for developed (*n* = 180) and validated (*n* = 61) models

Every model assessed was rated high risk of bias (Table 2; Fig. 2, precise screening question results explaining rationale for domain in Table S7, Figure S4). This comprised a median of 3/4 domains (Q1 to Q3: 2 to 3) rated high risk of bias in models developed, and 2/4 domains (Q1 to Q3: 1 to 3) for models validated. Whilst problematic analysis resulted in high risk of bias across all models, a post-hoc sensitivity analysis showed that 87% of models developed and 74% of models validated would have rated high risk of bias due to at least one other domain, even had analysis been adequate. There was no difference in proportion of models rated high risk of bias dependent upon type of machine learning methodology employed (Table S8), or when only the top-rated model from each article was included to calculate summary statistics (Table S9-S10).

**Table 2:** Individual question risk of bias ratings grouped as positive, negative or no information for developed and validated models

Title of domain /screening question		Developed models (*n* = 180) *n*, %			Validated models (*n* = 61) *n*, %		
**Overall risk of bias**		**LOW**	**HIGH**	**UNCLEAR**	**LOW**	**HIGH**	**UNCLEAR**
		*n* = 0, 0%	*n* = 180, 100%	*n* = 0, 0%	*n* = 0, 0%	*n* = 61, 100%	*n* = 0, 0%
Domain 1	**Participants**	**LOW**	**HIGH**	**UNCLEAR**	**LOW**	**HIGH**	**UNCLEAR**
		*n* = 48, 27%	*n* = 97, 54%	*n* = 35, 19%	*n* = 18, 29%	*n* = 39, 64%	*n* = 4, 7%
Screening questions:		**Yes /** **probably yes**	**No /** **probably no**	**No** **information**	**Yes /** **probably yes**	**No /** **probably no**	**No** **information**
1.1	Were appropriate data sources used, e.g., cohort, randomized controlled trial, or nested case-control study data?	*n* = 83, 46%	*n* = 79, 44%	*n* = 18, 10%	*n* = 43, 70%	*n* = 18, 30%	*n* = 0, 0%
1.2	Were all inclusions and exclusions of participants appropriate?	*n* = 62, 34%	*n* = 91, 51%	*n* = 27, 15%	*n* = 31, 51%	*n* = 27, 44%	*n* = 3, 5%
	51						
Domain 2	**Predictors**	**LOW**	**HIGH**	**UNCLEAR**	**LOW**	**HIGH**	**UNCLEAR**
		*n* = 48, 27%	*n* = 80, 44%	*n* = 52, 29%	*n* = 38, 62%	*n* = 16, 26%	*n* = 7, 12%
Screening questions:		**Yes /** **probably yes**	**No /** **probably no**	**No** **information**	**Yes /** **probably yes**	**No /** **probably no**	**No** **information**
2.1	Were predictors defined and assessed in a similar way for all participants?	*n* = 52, 29%	*n* = 110, 61%	*n* = 18, 10%	*n* = 37, 61%	*n* = 21, 34%	*n* = 3, 5%
2.2	Were predictor assessments made without knowledge of outcome data?	*n* = 123, 68%	*n* = 42, 23%	*n* = 15, 9%	*n* = 52, 85%	*n* = 2, 3%	*n* = 7, 12%
2.3	Are all predictors available at the time the model is intended to be used?	*n* = 168, 93%	*n* = 10, 6%	*n* = 2, 1%	*n* = 61, 100%	*n* = 0, 0%	*n* = 0, 0%
Domain 3	**Outcome**	**LOW**	**HIGH**	**UNCLEAR**	**LOW**	**HIGH**	**UNCLEAR**
		*n* = 71, 39%	*n* = 104, 58%	*n* = 5, 3%	*n* = 44, 72%	*n* = 9, 15%	*n* = 8, 13%
Screening questions:		**Yes /** **probably yes**	**No /** **probably no**	**No** **information**	**Yes /** **probably yes**	**No /** **probably no**	**No** **information**
3.1	Was the outcome determined appropriately?	*n* = 92, 51%	*n* = 77, 43%	*n* = 11, 6%	*n* = 54, 88%	*n* = 6, 10%	*n* = 1, 2%
3.2	Was a prespecified or standard outcome definition used?	*n* = 169, 94%	*n* = 11, 6%	*n* = 0, 0%	*n* = 57, 93%	*n* = 3, 5%	*n* = 1, 2%
3.3	Were predictors excluded from the outcome definition?	*n* = 168, 93%	*n* = 12, 7%	*n* = 0, 0%	*n* = 58, 95%	*n* = 3, 5%	*n* = 0, 0%
3.4	Was the outcome defined and determined in a similar way for all participants?	*n* = 111, 62%	*n* = 50, 28%	*n* = 19, 10%	*n* = 53, 87%	*n* = 8, 13%	*n* = 0, 0%
3.5	Was the outcome determined without knowledge of predictor information?	*n* = 76, 42%	*n* = 48, 27%	*n* = 56, 31%	*n* = 38, 62%	*n* = 4, 7%	*n* = 19, 31%
3.6	Was the time interval between predictor assessment and outcome determination appropriate?	*n* = 91, 51%	*n* = 71, 39%	*n* = 18, 10%	*n* = 49, 80%	*n* = 10, 17%	*n* = 2, 3%
Domain 4	**Analysis**	**LOW**	**HIGH**	**UNCLEAR**	**LOW**	**HIGH**	**UNCLEAR**
		*n* = 0, 0%	*n* = 180, 100%	*n* = 0, 0%	*n* = 0, 0%	*n* = 61, 100%	*n* = 0, 0%
Screening questions:		**Yes /** **probably yes**	**No /** **probably no**	**No** **information**	**Yes /** **probably yes**	**No /** **probably no**	**No** **information**
4.1	Were there a reasonable number of participants with the outcome?	*n* = 87, 48%	*n* = 62, 35%	*n* = 31, 17%	*n* = 42, 69%	*n* = 19, 31%	*n* = 0, 0%
4.2	Were continuous and categorical predictors handled appropriately?	*n* = 38, 21%	*n* = 122, 68%	*n* = 20, 11%	*n* = 53, 87%	*n* = 7, 11%	*n* = 1, 2%
4.3	Were all enrolled participants included in the analysis?	*n* = 0, 0%	*n* = 144, 80%	*n* = 36, 20%	*n* = 7, 11%	*n* = 37, 61%	*n* = 17, 28%
4.4	Were participants with missing data handled appropriately?	*n* = 13, 7%	*n* = 133, 74%	*n* = 34, 19%	*n* = 2, 3%	*n* = 38, 62%	*n* = 21, 35%
4.5	Was selection of predictors based on univariable analysis avoided?	*n* = 91, 51%	*n* = 62, 34%	*n* = 27, 15%			
4.6	Were complexities in the data (e.g. censoring, competing risks, sampling of control participants) accounted for appropriately?	*n* = 38, 21%	*n* = 117, 65%	*n* = 25, 14%	*n* = 8, 13%	*n* = 47, 77%	*n* = 6, 10%
4.7	Were relevant model performance measures evaluated appropriately?	*n* = 21, 12%	*n* = 159, 88%	*n* = 0, 0%	*n* = 4, 7%	*n* = 57, 93%	*n* = 0, 0%
4.8	Were model overfitting and optimism in model performance accounted for?	*n* = 27, 15%	*n* = 114, 63%	*n* = 39, 22%			
4.9	Do predictors and their assigned weights in the final model correspond to the results from the reported multivariable analysis?	*n* = 31, 17%	*n* = 17, 10%	*n* = 132, 73%			
Sensitivity analysis:		**LOW**	**HIGH**	**UNCLEAR**	**LOW**	**HIGH**	**UNCLEAR**
*Risk of bias rating not including analysis domain*:		*n* = 19, *11%*	*n* = 157, *87%*	*n* = 4, *2%*	*n* = 13, *21%*	*n* = 45, *74%*	*n* = 3, *5%*

* = 3% unclear risk of bias in domain 3 of developed models

#### Domain 1: participants

Participant selection contributed high risk of bias in 54% of developed models, and unclear risk of bias in 19%. Validated models were high risk in 64% and unclear in 7%. The screening question most frequently highlighting problems was 1.2 referring to the suitability of eligibility criteria, rated as ‘N/PN’ in 51% of developed models and 44% of validated models.

#### Domain 2: predictors

Predictor measurement contributed high risk of bias in 44% of developed models, and 26% in models validated. Question 2.1 assessing heterogeneity in the way predictors were defined and assessed across participants most frequently highlighted problems, rated as ‘N/PN’ in 61% of models developed and 34% of models validated.

#### Domain 3: outcome

Outcome measurement contributed high risk of bias in 58% of developed models, and 15% in models validated. Question 3.1 assessing appropriate determination of the outcome most frequently highlighted problems, 43% of models developed and 10% of models validated rated ‘N/PN’. The second most frequent was 3.6 pertaining to appropriate time between predictor and outcome assessment, where 39% of developed models and 16% of validated models were rated ‘N/PN’. Heterogeneity in the assessment of the outcome was a problem in 28% of models developed.

#### Domain 4: analysis

Every model developed and validated was rated as high risk of bias due to problematic analysis. Question 4.7 regarding model performance measures used to evaluate models was most frequently highlighted, inappropriate in 88% of models developed and 93% of those validated. Next most frequently flagged questions were 4.3 pertaining to all enrolled participants being included in the analysis, and 4.4 whether missing data was handled appropriately. In these questions 80% and 74% were rated ‘N/PN’ for developed models, and 61% and 62% for validated models respectively. Across all questions in domain 4, there was a median of 7.5/9 (Q1 to Q3: 6 to 8) questions answered ‘N/PN/NI’ in developed models and a median of 4/6 (Q1 to Q3: 4 to 5) in models validated.

## Discussion

We conducted a systematic review assessing the risk of bias within the development and validation of models to predict height and weight in fetal or paediatric medicine, and found all models assessed to be at high risk of bias, all having problematic analysis. Less than a third of models were reported to facilitate external validation. This highlights the importance of tools to assess the risk of bias of prediction models prior to application in clinical practice, and the necessity to improve understanding in prediction modelling to increase research impact.

### Selection and measurement of variables

Both height and weight are continuous outcomes, yet the most common model development method employed was logistic regression, alongside a variety of machine learning methods that predict categorical outcomes. Mishandling of continuous and categorical predictors was seen in over two-thirds of models developed, with inappropriate preferences to categorise continuous predictors, reducing power and introducing bias. This reproduces recent findings about logistic regression prediction models [35], despite open access guidelines advising against such practice [36, 37].

The assessment of predictors or outcomes was frequently heterogenous, particularly in multicentre or lengthy longitudinal studies. In over a third of models developed, there were limitations identified in the interval chosen between predictors and outcome of interest being too short or too long for clinical utility, and in 6% of models developed the predictors would not be available at the time the model was designed to be used.

### Sample size

Sample size consideration was rare, in keeping with broader reviews showing consistently inappropriate samples employed to develop prediction models [38]. Due to routine inclusion of multiple interaction terms and categorisation of predictors, machine learning models require larger samples to avoid overfitting [34]. Future research can benefit from published advice and software packages to assist calculating appropriate sample sizes [39].

### Analysis

The most frequent problems in analysis included using inappropriate performance measures, not accounting for all participants studied, and failures in handling missing data. Missing data mechanisms should be considered before carrying out complete case analysis. The exclusion of participants because of information only available at a stage *after* the prediction model is employed is inappropriate [40]. Multiple imputation offers the opportunity to account for missing values by using auxiliary variables and reduces the likelihood of bias, yet was rare, and when employed was often on a small subsection following listwise deletion [23, 41].

Only 16% of models assessed reported calibration alongside discrimination. Exclusively using calibration to assess predictions of continuous measurements may be appropriate, but over half reported only discrimination. Calibration plots to assess the accuracy of predictions across the spectrum of outcomes were rarely employed. Receiver operated characteristics (ROC) curves featured frequently, providing little insight beyond their area under the curve.

A lack of reporting of the final prediction model, as either an equation, code or online calculator for over 2/3 of models assessed calls into question the value of publishing such models. This was highlighted when assessing whether weights of variables in multivariable analysis were consistent with weights applied within the reported prediction model, a question that cannot be answered without transparent reporting of the final model itself.

Notwithstanding data analysis, 87% of developed models and 74% of models validated rated a high risk of bias based on the scoring of other domains. There would therefore remain significant problems with the underlying data available to the authors of most of these studies that means any model redevelopment, even with gold standard analysis, would remain at risk of bias. Instead, incorporating prediction model intentions within study designs and well defined protocols that promote transparency should be the focus of future research, to increase the likelihood of models that have the potential for clinical application [42].

### Clinical and research implications

We have shown a high risk of bias in predicting fundamental metrics used for clinical assessment in obstetrics and paediatrics, similar to that recently seen in infectious diseases [43], oncology [44], intensive care [45] and surgery [46], as well as across supervised learning methods in general [47]. Existing models most frequently used for comparison within studies related to obstetrics were the Hadlock formulae for fetal weight prediction developed in the 1980’s [9], formulae that have recently also shown superiority in meta-analysis of prediction for estimated fetal weight [48, 49]. Whilst failure to adopt novel models into clinical practice may in some cases reflect inertia within healthcare, our work alongside these meta-analyses highlights that despite exponential development of prediction models, there is a lack of high quality models developed that are suitable for clinical practice. In terms of paediatric studies predicting height, Bayley-Pinneau and Tanner’s formulae from the 1970’s and earlier were frequently used for comparison [13, 15], rather than the more recent 2011 UK model that adjusts for regression to the mean [16], or more modern open-source software that predicts height using bone age [17, 50]. When comparing newly developed models in future research, it is important that authors compare models to their most contemporaneous equivalents that are used within clinical practice. A lack of recent meta-analysis robustly comparing prediction models of adult height in children is an area for future research, although the heterogeneity in the predictors used for development of such models within our assessments highlights some of the challenges in direct model comparison. To maximise the likelihood of clinical application, we advocate for the application of high quality, robust prediction model strategies rather than the current trend of simply a greater quantity.

### Strengths and limitations

Most studies that validated an external model did not report external validation as their primary aim. Nonetheless, when analysis is used to conclude about the validity of the application of a model, or to advocate for another newly developed model, it is vital it is done robustly. Clinicians should critically analyse prediction models of height and weight using the updated PROBAST + AI [51], as we have seen significant limitations within model validation, that can result in model accuracy being interpreted incorrectly.

This review has provided a contemporary assessment of the risk of bias in models designed to predict height and weight, but is limited to those published in 2022, and thus some models produced more recently may be at lower risk of bias. We have not directly assessed the risk of bias that may exist within the original model development of those that are currently used in clinical practice, and have not included models published within the grey literature which limits the scope of this research. The studies included were heterogenous, some assessing growth over months or years, some assessing point estimates of height or weight using proxy variables. Whilst PROBAST and the more recent PROBAST + AI are well-structured tools that direct robust assessment of models, there remains an element of subjectivity in specific ratings. However, the independent rating by two authors and arbitration of disagreements by a medical statistician, alongside the large number of screening questions and domains that indicated a high risk of bias reassures that the overall risk of bias within all of the models assessed is high, and that their application in clinical practice cannot be recommended.

## Conclusions

Recent models developed and validated in 2022 to predict height and weight in fetuses and children are at high risk of bias and unsuitable for clinical practice. Until the standard of prediction modelling is improved, clinicians should continue to use traditional growth charts and simple formulae recommended by national and international bodies [10, 25–27]. The current focus on developing brand new models and use of opaque machine learning methodologies that lead to difficulties in interpretation should be redirected into external validation of existing models and incremental model updating [20, 52, 53].

## Supplementary Information

Supplementary Material 1.

## Data Availability

All raw data collected about each study and model reported in this study is available via the following link (tinyurl.com/442f4bdz).

## Abbreviations

PROBAST: Prediction model Risk Of Bias ASsessment Tool
AI: Artificial Intelligence
TRIPOD-SRMA: Transparent reporting of multivariable prediction models for individual prognosis or diagnosis: checklist for systematic reviews and meta-analyses
PRISMA: Preferred Reporting Items for Systematic reviews and Meta-Analyses
