# Supplementary material for: Risk of bias in machine learning and statistical models to predict height or weight: a systematic review in fetal and paediatric medicine
Source: Diagn Progn Res. 2025 Dec 15;9:32. doi: 10.1186/s41512-025-00215-6 (PMC12703889; doi:10.1186/s41512-025-00215-6)
Supplement: Supplementary file 1 — Supplementary Material 1. [file 41512_2025_215_MOESM1_ESM.docx]

**Supplementary material for Risk of bias in machine learning and statistical models to predict height or weight: a systematic review in fetal and paediatric medicine**

**Table S1:**

Search conducted via EMBASE and MEDLINE via OVID on 27/4/2023.

Machine learning or modelling terms:

| 1 | Machine Learning/ |
| --- | --- |
| 2 | (machine adj1 (learn$ or model$)).ti,ab,kw. |
| 3 | Deep Learning/ |
| 4 | (deep adj2 learn$).ti,ab,kw. |
| 5 | exp Supervised Machine Learning/ |
| 6 | (supervised adj2 machine adj2 learn$).ti,ab,kw. |
| 7 | ((support or relevance) adj2 vector adj2 (machine$ or classification$)).ti,ab,kw. |
| 8 | "Neural Networks (Computer)"/ |
| 9 | (neural adj2 network$).ti,ab,kw. |
| 10 | ((statistical or "statistical-learning") adj1 (learn$ or strateg$)).ti,ab,kw. |
| 11 | (multi adj2 layer adj1 perceptron$).ti,ab,kw. |
| 12 | (random adj2 forest$).ti,ab,kw. |
| 13 | "RF classifi$".ti,ab,kw. |
| 14 | (lasso or ridge or kernel or ensemble or bagging or bagged or bootstrap$ or boosting or boosted or fuzzy).ti,ab,kw. |
| 15 | ((penali?ed or regulari?ed) adj2 ('likelihood' or 'regression' or 'logistic' or 'survival' or 'estimat$' or 'function$' or 'method$' or 'least' or 'ensemble')).ti,ab,kw. |
| 16 | ((classification or regression or estimation or decision) adj2 tree$).ti,ab,kw. |
| 17 | (bayes$ adj1 network$).ti,ab,kw. |
| 18 | (nearest adj1 neighbo?r).ti,ab,kw. |
| 19 | (k-nearest adj1 neighbo?r).ti,ab,kw. |
| 20 | (elastic adj1 net).ti,ab,kw. |
| 21 | (naive adj1 bayes$).ti,ab,kw. |
| 22 | ((nonparametric or "non-parametric") adj2 (model$ or analys$)).ti,ab,kw |
| 23 | (KNN or ANN or ANNs or RNN or RF or SVM or NB or CART or DT or MLP).ti,ab,kw. |
| 24 | Logistic Models/ |
| 25 | (logistic adj4 (model$ or regression)).ti,ab,kw. |
| 26 | Linear Models/ |
| 27 | (linear adj2 (model$ or regression)).ti,ab,kw. |
| 28 | (proportion$ adj2 odds adj2 regression).ti,ab,kw. |
| 29 | Least-Squares Analysis/ |
| 30 | (least adj2 square$).ti,ab,kw. |
| 31 | Survival Analysis/ |
| 32 | (survival adj1 (analys$ or model$)).ti,ab,kw. |
| 33 | Proportional Hazards Models/ |
| 34 | (proportional adj1 hazard$).ti,ab,kw. |
| 35 | ((cox or parametric) adj1 (regression or model$)).ti,ab,kw. |
| 36 | (semi adj2 parametric adj1 (regression or model$)).ti,ab,kw. |
| 37 | Disease-Free Survival/ |
| 38 | Progression-Free Survival/ |
| 39 | ((disease or progression or event) adj2 free adj1 survival).ti,ab,kw. |
| 40 | (overall adj1 survival).ti,ab,kw. |
| 41 | formula$.ti,ab,kw. |
| 42 | equation$.ti,ab,kw. |
| 43 | "curve matching".ti,ab,kw. |
| 44 | "interpolation method$".ti,ab,kw. |

Performance terms:

| 45 | Prognosis/ |
| --- | --- |
| 46 | (prognos$ adj1 (modelling or modeling or model or models or predict$ or index or performance or nomogram or tools or ability or accuracy or probability or risk or factor$ or marker$ or biomarker$)).ti,ab,kw. |
| 47 | "risk model$".ti,ab,kw. |
| 48 | "predict$ the prognosis of".ti,ab,kw. |
| 49 | "predict$ the risk of".ti,ab,kw. |
| 50 | "predict$ the probability of".ti,ab,kw. |
| 51 | Probability/ |
| 52 | (probability adj1 (modelling or modeling or model or models)).ti,ab,kw. |
| 53 | predict$ adj3 (modelling or modeling or model or models or nomogram or tools or performance or ability or index or accuracy or probability or risk or factor$ or marker$ or biomarker$).ti,ab,kw. |
| 54 | "candidate predictor$".ti,ab,kw. |
| 55 | "predictive clinical parameter$".ti,ab,kw. |
| 56 | ((discrimination or discriminative or discriminatory) adj1 (accuracy or ability or performance or value or model or models or power or capacity or capabilit$ or efficiency)).ti,ab,kw. |
| 57 | (discriminability or c-index or c-statistic or concordance or DCA).ti,ab,kw. |
| 58 | "decision curve".ti,ab,kw. |
| 59 | (calibrat$ adj1 (plot$ or curve$ or slope$ or model or models)).ti,ab,kw. |
| 60 | (brier adj1 score$).ti,ab,kw. |
| 61 | (performance adj1 (classification or classifier or clinical or accuracy or validation or metrics or diagnostic or AUC)).ti,ab,kw. |
| 62 | (sensitivity or specificity or PPV or NPV).ti,ab,kw. |
| 63 | "correctly classified".ti,ab,kw. |
| 64 | "clinical accuracy".ti,ab,kw. |
| 65 | "positive predictive value$".ti,ab,kw. |
| 66 | "negative predictive value$".ti,ab,kw. |
| 67 | (classification or classifier).ti,ab,kw. |
| 68 | Area Under Curve/ |
| 69 | "Area under the curve".ti,ab,kw. |
| 70 | "Area under the ROC curve".ti,ab,kw. |
| 71 | "Area under the ROC".ti,ab,kw. |
| 72 | "Area Under the Receiver Operat$ Characteristic$".ti,ab,kw. |
| 73 | ROC Curve/ |
| 74 | "receiver operating characteristic$".ti,ab,kw. |
| 75 | (ROC or AUC or AUROC).ti,ab,kw. |
| 76 | "Hosmer-Lemeshow".ti,ab,kw. |
| 77 | "H-L test".ti,ab,kw. |
| 78 | "expected ratio".ti,ab,kw. |
| 79 | "observed ratio".ti,ab,kw. |
| 80 | "E:O ratio".ti,ab,kw. |
| 81 | r?squared.ti,ab,kw. |
| 82 | MSE$.ti,ab,kw. |
| 83 | Correlation.ti,ab,kw. |
| 84 | risk accumulation$.ti,ab,kw. |
| 85 | "root mean square$ error".ti,ab,kw. |
| 86 | "root mean square$ percentage error".ti,ab,kw. |
| 87 | RMSE$.ti,ab,kw. |
| 88 | explained varia$.ti,ab,kw. |
| 89 | error.ti,ab,kw. |
| 90 | ("F?score" or "F score" or "F1 score" or "F2 score" or "F-1 score" or "F-2 score").ti,kw,ab. |
| 91 | ("F?measure" or "f measure" or "f1 measure" or "f2 measure" or "f?1 measure" or "f?2 measure").ti,kw,ab. |
| 92 | r?2.ti,ab,kw. |
| 93 | recall.ti,ab,kw. |
| 94 | precision.ti,ab,kw. |
| 95 | accuracy.ti,ab,kw. |
| 96 | "detection rate".ti,ab,kw |
| 97 | "false positive rate".ti,ab,kw |
| 98 | "hamming loss".ti,ab,kw. |
| 99 | "jaccard score".ti,ab,kw. |
| 100 | "jaccard index".ti,ab,kw. |
| 101 | "youden score".ti,ab,kw. |
| 102 | "youden index".ti,ab,kw. |

Growth terms

| 103 | growth$.ti,ab,kw. |
| --- | --- |
| 104 | height$.ti,ab,kw. |
| 105 | weight$.ti,ab,kw. |
| 106 | fat$.ti,ab,kw. |
| 107 | obes$.ti,ab,kw |
| 108 | macrosom$.ti,ab,kw. |
| 109 | stunt$.ti,ab,kw. |
| 110 | stature$.ti,ab,kw. |
| 111 | "failure to thrive".ti,ab,kw. |

Patient demographic terms

| 112 | (adolescen$ or child$ or infan$ or neonat$ or neo-nat$ or born$ or paediatric$ or peadiatric$ or pediatric$ or perinat$ or fetus$ or foetus$ or fetal$ or foetal$).ti,ab,kw. |
| --- | --- |
| 113 | adolescent/ or child/ or infant/ or fetus/ |

Combination of terms

| 114 | Or/1-43 | Machine learning or modelling terms |
| --- | --- | --- |
| 115 | Or/44-92 | Performance terms |
| 116 | Or/93-99 | Growth terms |
| 117 | Or/100-101 | Adolescent, Infant, Child or fetus terms |
| 118 | And/102-105 | **Systematic Review Collection of terms** |
| 119 | limit 118 to yr="2022" | **Time restriction** |
| 120 | limit 119 to (english) | **Journal article and language restriction** |
| 121 | Deduplicate 120 | **Final titles and abstracts to screen** |

**Table S2:**

PROBAST domains and signalling questions used for data extraction (1, 2).

| **PROBAST domain and signalling questions** | **Notes** |
| --- | --- |
| 1. **PARTICIPANTS** |  |
| - 1. *Were appropriate data sources used, e.g., cohort, randomized controlled trial, or nested case–control study data?* |  |
| - 1. *Were all inclusions and exclusions of participants appropriate?* |  |
| PREDICTORS |  |
| - 1. *Were predictors defined and assessed in a similar way for all participants?* |  |
| - 1. *Were predictor assessments made without knowledge of outcome data?* |  |
| - 1. *Are all predictors available at the time the model is intended to be used?* |  |
| 1. **OUTCOMES** |  |
| - 1. *Was the outcome determined appropriately?* |  |
| - 1. *Was a prespecified or standard outcome definition used?* |  |
| - 1. *Were predictors excluded from the outcome definition?* |  |
| - 1. *Was the outcome defined and determined in a similar way for all participants?* |  |
| - 1. *Was the outcome determined without knowledge of predictor information?* |  |
| - 1. *Was the time interval between predictor assessment and outcome determination appropriate?* |  |
| 1. **ANALYSIS** |  |
| - 1. *Were there a reasonable number of participants with the outcome?* |  |
| - 1. *Were continuous and categorical predictors handled appropriately?* |  |
| - 1. *Were all enrolled participants included in the analysis?* |  |
| - 1. *Were participants with missing data handled appropriately?* |  |
| - 1. *Was selection of predictors based on univariable analysis avoided?* |  |
| - 1. *Were complexities in the data (e.g., censoring, competing risks, sampling of control participants) accounted for appropriately?* | Not applicable to assessment of model validation |
| - 1. *Were relevant model performance measures evaluated appropriately?* |  |
| - 1. *Were model overfitting and optimism in model performance accounted for?* | Not applicable to assessment of model validation |
| - 1. *Do predictors and their assigned weights in the final model correspond to the results from the reported multivariable analysis?* | Not applicable to assessment of model validation |

**Table S3:**

Study specific guidance for PROBAST ratings within each signalling question

| **Domain 1: Participants** |
| --- |
| **1.1 Were appropriate data sources used, e.g. cohort, RCT or nested case-control study?** |
| **Factors decreasing risk of bias (Y/PY)**   - prospective longitudinal cohorts (RCT or proper registry) with consistent methods for participant inclusion and exclusion – predefined predictors and outcome determination - case control/cohort studies are low risk of bias if the original cohort/registry outcome frequency is adjusted for. Look for ‘reweighting’ or ‘inverse sampling fraction’ of the outcome.   **Factors increasing risk of bias (N/PN)**   - existing cohorts with potentially inconsistent participant inclusion/exclusion criteria – data collected for other purposes than developing and validating a prediction model (if a protocol is given this may reduce the risk of bias) - RCTs have narrower eligibility for participants, they also need to ensure treatment is included as a predictor, if not this is high risk of bias - ill-defined case-control/cohort studies are at high risk of bias - non-nested case-control design   *Notes:*  *Studies using questionnaire data are not necessarily at high risk of bias and are considered on a case by case basis. If the population is appropriately representative or weighted to make itself representative of the population in which it could be employed their use can be justified.* |
| **1.2 Were all inclusions and exclusions of participants appropriate?** |
| **Factors decreasing risk of bias (Y/PY)**   - inclusion/exclusion appropriate to get representative sample of target population - participants correspond to unselected participants of interest   **Factors increasing risk of bias (N/PN)**   - inappropriate inclusion/exclusion of participants e.g., subgroups of populations that are not representative of the target population - includes participants who have already had the outcome e.g., including pre-operative transfusion patient when predicting intra- or post-operative transfusion or if the blood transfusion outcome is a self-reported outcome measure   *Notes:*  *Inclusion and exclusion criteria should not include any attribute that is retrospectively applied, and cannot be known at the time of entry into the study (e.g. excluding babies born prematurely). Where studies exclude patients on the basis of missing data, this is considered within the analysis rating, not the domain of participants.* |
|  |
| **Domain 2: Predictors** |
| **2.1 Were predictors defined and assessed in a similar way for all participants?** |
| **Factors decreasing risk of bias (Y/PY)**   - predictors defined and assessed in the same way - definitions of predictors and their assessment were similar for all participants   **Factors increasing risk of bias (N/PN)**   - predictors not defined and assessed in the same way - predictors involving subjective judgement/assessment or skilled training, relying on the ability of the assessor - data from multiple sources likely to have used different definitions   *Notes:*  *Where birth weight is defined without reference to precise equipment, this is not considered a high risk of bias due to the simplicity of the measurement, provided it was conducted in hospital* |
| **2.2 Were predictor assessments made without knowledge of outcome data?** |
| **Factors decreasing risk of bias (Y/PY)**   - outcome information was stated as not used during predictor assessment or was clearly not (yet) available to those assessing predictors - blinding of the outcome   **Factors increasing risk of bias (N/PN)**   - clear that outcome information was used when assessing predictors - retrospectively recorded predictors   *Notes:*  *Whilst blinding is to be considered, cases that do not specifically report blinding of the outcome can be considered on a chronological basis (e.g. ultrasonographic fetal measurements will be made at time of scan, and can be rated as PY, as birth weight cannot be known at the time)* |
| **2.3 Are all predictors available at the time the model is intended to be used?** |
| **Factors decreasing risk of bias (Y/PY)**   - *for development studies,* the model can be used in the real world and predictors are available in clinical settings - included predictors would be available at the time the model is intended to be used for prediction (e.g., if values from umbilical cord blood is used to predict the baby’s weight, this would not be useful in clinical practice because umbilical cord blood is only taken after the baby is born)   **Factors increasing risk of bias (N/PN)**   - *for validation studies,* predictor data needed for the model is missing from the validation dataset - predictors would not be available at the time the model is intended to be used for prediction |
|  |
| **Domain 3: Outcome** |
| **3.1 Was the outcome determined appropriately?** |
| **Factors decreasing risk of bias (Y/PY)**   - method of outcome determination has been used which is considered optimal or acceptable by guidelines or previous publications on the topic   **Factors increasing risk of bias (N/PN)**   - a clearly suboptimal method has been used that causes unacceptable error in determining outcome status in participants   *Notes:*  *Where birth weight is defined without reference to precise equipment, this is not considered a high risk of bias due to the simplicity of the measurement, provided it was conducted in hospital* |
| **3.2 Was a prespecified or standard outcome definition used?** |
| **Factors decreasing risk of bias (Y/PY)**   - pre-specified/standard objective outcome - substantiated from clinical guidelines/previous studies/available protocol - prespecified categories are used to group outcomes   **Factors increasing risk of bias (N/PN)**   - composite outcomes - uses atypical thresholds, or creates multiple thresholds on continuous outcome - outcomes created from the same dataset (e.g. via cluster analysis) |
| **3.3 Were predictors excluded from the outcome definition?** |
| **Factors decreasing risk of bias (Y/PY)**   - outcome determined without any predictor information   **Factors increasing risk of bias (Y/PY)**   - ≥1 of the predictors forms part of the outcome definition   *Notes:*  *Using previous height or weight to predict future height or weight is sensible and not considered as the same predictor information. However, if current height or weight is used within the creation of another metric (e.g. height or weight trajectory) that is then used as a predictor, the model is at a high risk of bias* |
| **3.4 Was the outcome defined and determined in a similar way for all participants?** |
| **Factors decreasing risk of bias (Y/PY)**   - outcomes were defined and determined in a similar way for all participants   **Factors increasing risk of bias (N/PN)**   - data collected for non-research purposes e.g., routinely collected data from registries   *Notes:*  *Where birth weight is defined without reference to precise equipment, this is not considered a high risk of bias due to the simplicity of the measurement, provided it was conducted in hospital* |
| **3.5 Was the outcome determined without knowledge of predictor information?** |
| **Factors decreasing risk of bias (Y/PY)**   - predictor information was not known when determining the outcome status - outcome status determination is clearly reported as determined without knowledge of predictor information   **Factors increasing risk of bias (N/PN)**   - predictor information was used when determining the outcome status - outcomes were clearly defined and determined in a different way for some participants - outcomes requiring interpretation - predictor information would be available at time of outcome determination (consider the potential consequences)   *Notes:*  *Due to the objective nature of measuring height and weight, it is important to consider the context of the data collection prior to rating a model at high risk of bias due to a lack of information or failure to blind those measuring the outcome to predictor information* |
| **3.6 Was the time interval between predictor assessment and outcome determination appropriate?** |
| **Factors decreasing risk of bias (Y/PY)**   - time interval between predictor assessment and outcome determination was appropriate to enable suitable assessment of the predictive accuracy   **Factors increasing risk of bias (N/PN)**   - time between predictor assessment and outcome determination is too long/too short to enable suitable assessment of the predictive accuracy   *Notes:*  *Metrics reported from models developed or applied to changes in height or weight very soon after predictor assessment are likely to show inflated accuracy. This must be considered in the clinical context of when the prediction model is designed to be applied. For instance, a third trimester ultrasound to predict birthweight may be conducted late in pregnancy close to when birthweight is attained. However, if the model is designed to be employed to inform about the decision to proceed to caesarean section, then this has valid clinical utility to be applied at that point in pregnancy, and is therefore not necessarily at risk of bias.* |
|  |
| **Domain 4: Analysis** |
| **4.1 Were there a reasonable number of participants with the outcome?** |
| **Factors decreasing risk of bias (Y/PY)**   - *for model development studies,* if the outcome is categorical within the model, the number of participants with the outcome relative to the number of candidate predictor parameters is ≥20 (events per variable ≥20)*. If the outcome is continuous, consider a number of participants >100 as a minimum - *for model validation studies,* if the number of participants with the outcome is ≥100, or participants>100 for continuous outcomes   **Factors increasing risk of bias (N/PN)**   - *for model development studies,* if the number of participants with the outcome relative to the number of candidate predictor parameters is <10)* , or number of participants <100 for a continuous outcome - *for model validation studies,* if the number of participants with the outcome is <100, or participants<100 for continuous outcomes   *Notes:*  **For events per variable between 10 and 20, the item should be rated as either probably yes or probably no, depending on the outcome frequency, overall model performance, and distribution of the predictors in the model*  *Consider the number of degrees of freedom used by categorical predictors. For instance, if a predictor is ethnicity that contains 3 categories of ‘white’, ‘black’ and ‘other’, this will use 2 degrees of freedom and require the estimation of 2 candidate predictor parameters. Extra parameters are estimated if there are extra terms added to assess non-linearity (e.g. quadratic terms used two degrees of freedom to estimate βx + βx^2^).*  *Consider what parameter the model is estimating prior to considering events per variable. If the model itself is predicting a continuous outcome, but internal validation assesses those outcomes against a standardised dichotomous threshold, the model sample size should be considered against the continuous variable that it is assessing, not the number of categorical results above the threshold. However, if there are few results over or under a clinically significant threshold, this must be considered within domain 1 as a potential risk of bias due to a bias sample.* |
| **4.2 Were continuous and categorical handled appropriately?** |
| **Factors decreasing risk of bias (Y/PY)**   - continuous kept as continuous predictors - continuous predictors examined for nonlinearity – look for ‘fractional polynomials’ or ‘restricted cubic splines’   **Factors increasing risk of bias (N/PN)**   - continuous predictors are dichotomised - continuous predictors are categorised, especially using widely accepted clinical cut-offs**,** data driven cut-offs increase the risk of bias - *for validation studies,* predictors are collected using different formats |
| **4.3 Were enrolled participants included in the analysis?** |
| **Factors decreasing risk of bias (Y/PY)**   - all participants enrolled in the study are included in the data analysis, or a low number are excluded   **Factors increasing risk of bias (N/PN)**   - some or a subgroup of participants are inappropriately excluded from the analysis, including participants with ‘unclear’ findings, missing data, or outliers |
| **4.4 Were participants with missing data handled appropriately?** |
| **Factors decreasing risk of bias (Y/PY)**   - no missing values of predictors or outcomes and the study explicitly reports that participants are not excluded based on missing data - missing values are handled using multiple imputation - comparing results with and without missing data   **Factors increasing risk of bias (N/PN)**   - missing data are omitted from the analysis - method of handling missing data is clearly flawed, e.g., missing indicator method or inappropriate use of last value carried forward - study had no explicit mention of methods to handle missing data   *Notes:*  *If missing data information not reported then assume complete case analysis was conducted* |
| **4.5 Was selection of predictors based on univariable analysis avoided?** |
| **Factors decreasing risk of bias (Y/PY)**   - predictors are not selected based on univariable analysis prior to multivariable modelling - predictors selected on existing knowledge, they are reliable, consistent, applicable, available, and credible - credible/a-priori predictors are forced into the model   **Factors increasing risk of bias (N/PN)**   - predictors are selected based on univariable analysis prior to multivariable modelling   *Notes:*  *This question applies for development studies only* |
| **4.6 Were complexities in the data (e.g., censoring, competing risks, sampling of control participants) accounted appropriately?** |
| **Factors decreasing risk of bias (Y/PY)**   - complexities in the data are accounted for appropriately - clear that any potential data complexities have been identified appropriately as unimportant - multilevel or random effects models for multiple outcome measures   **Factors increasing risk of bias (N/PN)**   - complexities in the data that could affect model performance are ignored   *Notes:*  *For a complexity in the data to apply, the criticism must not have already been considered in any of the other screening questions. If a dataset has no complexities that aren’t considered elsewhere, then this should be marked as ‘Y’ rather than ‘NI’* |
| **4.7 Were relevant model performance measures evaluated appropriately?** |
| **Factors decreasing risk of bias (Y/PY)**   - both calibration and discrimination* are evaluated appropriately (including relevant measures tailored for models predicting survival outcomes).   **Factors increasing risk of bias (N/PN)**   - both calibration and discrimination are not evaluated - only goodness-of-fit tests, such as the Hosmer–Lemeshow test, are used to evaluate calibration - if classification measures (like sensitivity, specificity, or predictive values) were presented using predicted probability thresholds derived from the data set at hand/non-clinical cut-offs   *Notes:*  *If model is predicting a continuous outcome without a clinically significant threshold associated with it, it may be appropriate to only have assessed calibration. However, if predicting a continuous outcome in relation to a threshold that would change a clinical decision, discrimination around that threshold should be assessed* |
| **4.8 Was model overfitting, underfitting, and optimism in model performance accounted for?** |
| **Factors decreasing risk of bias (Y/PY)**   - internal validation techniques, such as bootstrapping and cross-validation including all model development procedures, have been used to account for any optimism in model fitting, and subsequent adjustment of the model performance estimates have been applied   **Factors increasing risk of bias (N/PN)**   - no internal validation has been performed, or if internal validation consists only of a single random split-sample of participant data - bootstrapping or cross-validation did not include all model development procedures including any variable selection   *Notes:*  *This question applies for development studies only* |
| **4.9 Do predictors and their assigned weights in the final model correspond to the results from the reported multivariable analysis?** |
| **Factors decreasing risk of bias (Y/PY)**   - predictors and regression coefficients in the final model correspond to reported results from multivariable analysis   **Factors increasing risk of bias (N/PN)**   - predictors and regression coefficients in the final model do not correspond to reported results from multivariable analysis   *Notes:*  *If final model is not presented, or model code is not available via supplementary material, then rate as NI. If model is presented once, then one can assume there is no conflicting model parameters. High risk of bias is considered when there is conflicting model information presented in different places within the paper or supplementary material.* |

**Link to data collection proforma:**

https://tinyurl.com/3aazxyen

**Table S4:**

List of all included studies, study code to correlate to figure S4, the model types rated, and the total number of models assessed within each study

| **Title of study** | **Study code** | **Type of Study** | **No. of models rated** |
| --- | --- | --- | --- |
| Development and validation of a nomogram to predict poor short term response to recombinant human growth hormone treatment in children with growth disorders (3) | A003 | Development | 2 |
| Interpretable machine learning to identify important predictors of birth weight: A prospective cohort study (4) | A004 | Development | 8 |
| Curve matching to predict growth in patients receiving growth hormone therapy: An interpretable & explainable method (5) | A005 | Development | 1 |
| Machine learning algorithms for predicting low birth weight in Ethiopia (6) | A006 | Development | 8 |
| Study of Multidimensional and High-Precision Height Model of Youth Based on Multilayer Perceptron (7) | A007 | Development and external validation of the same model | 1 |
| Risk scores for predicting small for gestational age infants in Japan: The TMM BirThree cohort study (8) | A008 | Development | 3 |
| Prediction of late-onset fetal growth restriction using a combined first- and second-trimester screening model (9) | A010 | Development | 3 |
| Height Gain After Spinal Fusion for Idiopathic Scoliosis: Which Model Fits Best? (10) | A012 | Validation | 5 |
| A Predictive Model for Large-for-Gestational-Age Infants among Korean Women with Gestational Diabetes Mellitus Using Maternal Characteristics and Fetal Biometric Parameters (11) | A013 | Development of a model with validation of two already existing models | 3 |
| A predictive model of macrosomic birth based upon real world clinical data from pregnant women (12) | A014 | Development | 1 |
| A Machine Learning Based Intrauterine Growth Restriction (IUGR) Prediction Model for Newborns (13) | A015 | Development | 10 |
| Long-term effectiveness of growth hormone therapy in children born small for gestational age: An analysis of LG growth study data (14) | A016 | Development | 1 |
| The Effect of Risk Accumulation on Childhood Stunting: A Matched Case-Control Study in China (15) | A020 | Development | 1 |
| Establishment of a New Equation for Ultrasonographic Estimated Foetal Weight in Chongqing: A Prospective Study (16) | A022 | Development and external validation of the same model with validation of 3 other already existing models | 4 |
| First-trimester screening model for small-for-gestational-age using maternal clinical characteristics, serum screening markers, and placental volume: prospective cohort study (17) | A023 | Development | 1 |
| Derivation and assessment of a sex-specific fetal growth standard (18) | A025 | Development | 1 |
| Development and validation of nomogram for prediction of low birth weight: a large-scale cross-sectional study in northwest China (19) | A026 | Development | 1 |
| Antenatal prediction of fetal macrosomia in pregnancies affected by maternal pre-gestational diabetes (20) | A027 | Development | 1 |
| Prenatal prediction of very late onset small-for-gestational age newborns in low-risk pregnancies (21) | A028 | Development | 3 |
| Sonographic growth curves versus neonatal birthweight growth curves for the identification of fetal growth restriction (22) | A029 | Validation | 1 |
| Frequency of Correct Fetal Weight Estimation by Clinical and Ultrasound Methods in Pregnant Women (23) | A031 | Validation | 2 |
| Predicting risks of low birth weight in Bangladesh with machine learning (24) | A032 | Development | 2 |
| **Title** |  | **Type of Study** | **No. of models rated** |
| Analytical Comparison of Risk Prediction Models for the Onset of Macrosomia Based on Three Statistical Methods (25) | A035 | Development | 3 |
| Prediction of small-for-gestational-age neonates at 33-39 weeks' gestation in China: logistic regression modeling of the contributions of second- and third-trimester ultrasound data and maternal factors (26) | A036 | Development of 4 models with validation of 2 already existing models | 6 |
| Johnsons Technique versus Hadlock - A Comparative Study to Estimate Foetus Weight (27) | A037 | Validation | 2 |
| Estimated fetal weight accuracy in pregnancies with preterm prelabor rupture of membranes by the Hadlock method (28) | A038 | Validation | 1 |
| Personalized Model to Predict Small for Gestational Age at Delivery Using Fetal Biometrics, Maternal Characteristics, and Pregnancy Biomarkers: A Retrospective Cohort Study of Births Assisted at a Spanish Hospital (29) | A039 | Development of a model with validation of an already existing model | 2 |
| Investigation and Application of Risk Factors of Macrosomia Based on 10,396 Chinese Pregnant Women (30) | A040 | Development | 3 |
| Nomogram-based risk prediction of macrosomia: a case-control study (31) | A041 | Development | 1 |
| A Novel Method for Adult Height Prediction in Children With Idiopathic Short Stature Derived From a German-Dutch Cohort (32) | A043 | Development of 10 models with validation of 3 already existing models | 13 |
| Adult Height in Girls With Idiopathic Premature Adrenarche: A Cohort Study and Design of a Predictive Model (33) | A045 | Development | 2 |
| Birth weight prediction by Lee formula based on fractional thigh volume in term pregnancies: is it helpful? (34) | A046 | Validation | 1 |
| Dynamic prediction model of fetal growth restriction based on support vector machine and logistic regression algorithm (35) | A049 | Development | 8 |
| Performance of Machine Learning Classifiers in Classifying Stunting among Under-Five Children in Zambia (36) | A050 | Development | 5 |
| A multivariate analysis to propose linear models for the stature estimation in the Sabahan young adult population (37) | A051 | Development | 1 |
| Prediction of Neonatal Growth Restriction in Fetuses with Gastroschisis by Early Third Trimester Ultrasonography Utilizing Contemporary Birth Weight Percentiles (38) | A052 | Validation | 7 |
| The Percentage of Mature Height as a Morphometric Index of Somatic Growth: A Formal Scrutiny of Conventional Simple Ratio Scaling Assumptions (39) | A053 | Validation | 1 |
| Accuracy of the sonographic determination of estimated fetal weight in anhydramnios (40) | A054 | Validation | 1 |
| Analysis of risk factors and construction of a prediction model for short stature in children (41) | A058 | Development | 1 |
| Estimation and feasibility of correction modelling for mother-reported child height and weight at 2 years using data from the Australian CHAT trial (42) | A059 | Development | 6 |
| Prediction of large-for-gestational-age infant by fetal growth charts and hemoglobin A1c level in pregnancy complicated by pregestational diabetes (43) | A063 | Development of 3 models with validation of 1 already existing model | 4 |
| Weight Status of Children Participating in the National Spina Bifida Patient Registry (44) | A064 | Development | 1 |
| Correlation between estimated fetal weight and weight at birth in infants with gastroschisis and omphalocele (45) | A070 | Validation | 3 |
| Role of umbilicocerebral and cerebroplacental ratios in prediction of perinatal outcome in FGR pregnancies (46) | A078 | Development of 2 models with validation of 2 already existing models | 4 |
| Are body roundness index and a body shape index in the first trimester related to foetal macrosomia? (47) | A081 | Validation | 3 |
| **Title** |  | **Type of Study** | **No. of models rated** |
| The value of fetal growth biometry velocities to predict large for gestational age (LGA) infants (48) | A082 | Development of 4 models with validation of 1 already existing model | 5 |
| A prenatal standard for fetal weight improves the prenatal diagnosis of small for gestational age fetuses in pregnancies at increased risk (49) | A086 | Validation | 3 |
| The birth weight of macrosomia influence the accuracy of ultrasound estimation of fetal weight at term (50) | A091 | Validation | 1 |
| Predicting height from ulna length for the determination of weight status in New Zealand adolescents: A cross-sectional study (51) | A099 | Development of 1 model with validation of 2 already existing models | 3 |
| Maternal Body Mass Index, Early-Pregnancy Metabolite Profile, and Birthweight (52) | A108 | Development | 3 |
| Identifying factors associated with central obesity in school students using artificial intelligence techniques (53) | A109 | Development | 11 |
| Predicting South Korean adolescents vulnerable to obesity after the COVID-19 pandemic using categorical boosting and shapley additive explanation values: A population-based cross-sectional survey (54) | A117 | Development | 1 |
| Validity of Scottish predictors of child obesity (age 12) for risk screening in mid-childhood: a secondary analysis of prospective cohort study data-with sensitivity analyses for settings without various routinely collected predictor variables (55) | A118 | Development | 2 |
| Predicting risk of overweight or obesity in Chinese preschool-aged children using artificial intelligence techniques (56) | A120 | Development | 11 |
| Predicting the earliest deviation in weight gain in the course towards manifest overweight in offspring exposed to obesity in pregnancy: a longitudinal cohort study (57) | A121 | 2 models with development and external validation of the same models, alongside 1 other model developed | 3 |
| Development of a nutritional risk screening tool for preterm children in outpatient settings during a complementary feeding period: a pilot study (58) | A133 | 3 models with development and external validation of the same models | 3 |
| Prediction of pre-eclampsia complicated by fetal growth restriction and its perinatal outcome based on an artificial neural network model (59) | A134 | Development | 1 |
| Integrating longitudinal clinical and microbiome data to predict growth faltering in preterm infants (60) | A135 | Development | 6 |
| Comparing fetal biometric growth velocity versus estimated fetal weight for prediction of neonatal small for gestational age (61) | A136 | Development of 1 model with validation of 3 already existing models | 4 |
| Application of Machine Learning Approaches to Predict Postnatal Growth Failure in Very Low Birth Weight Infants (62) | A137 | Development | 25 |
| Comparing Attained Weight and Weight Velocity during the First 6 Months in Predicting Child Undernutrition and Mortality (63) | A139 | Validation | 3 |
| Prediction of Low Birth Weight by Quadruple Parameters in High-Risk Pregnancies (64) | A144 | Development | 1 |
| Vitamin D Deficiency, Excessive Gestational Weight Gain, and Oxidative Stress Predict Small for Gestational Age Newborns Using an Artificial Neural Network Model (65) | A145 | Development | 1 |
| High-risk Growth Trajectory Related to Childhood Overweight/Obesity and Its Predictive Model at Birth (66) | A146 | Development | 5 |

**Table S5:**

Countries from which the participants used in articles were assessed

| **Country** | **n** |
| --- | --- |
| Australia | 1 |
| Bangladesh | 1 |
| Brazil / Italy / Oman / UK / USA | 1 |
| China | 20 |
| Ethiopia | 1 |
| Germany | 4 |
| India | 3 |
| Ireland | 1 |
| Israel | 1 |
| Italy | 1 |
| Japan | 1 |
| Malaysia | 1 |
| Mexico | 1 |
| Netherlands | 2 |
| New Zealand | 1 |
| Pakistan | 1 |
| Poland | 1 |
| Qatar | 1 |
| Scotland | 1 |
| South Korea | 5 |
| Spain | 3 |
| Tanzania | 1 |
| Turkey | 1 |
| USA | 8 |
| USA & UK | 1 |
| Zambia | 1 |

**Figure S1:**

Map of countries from which participant data was used in studies


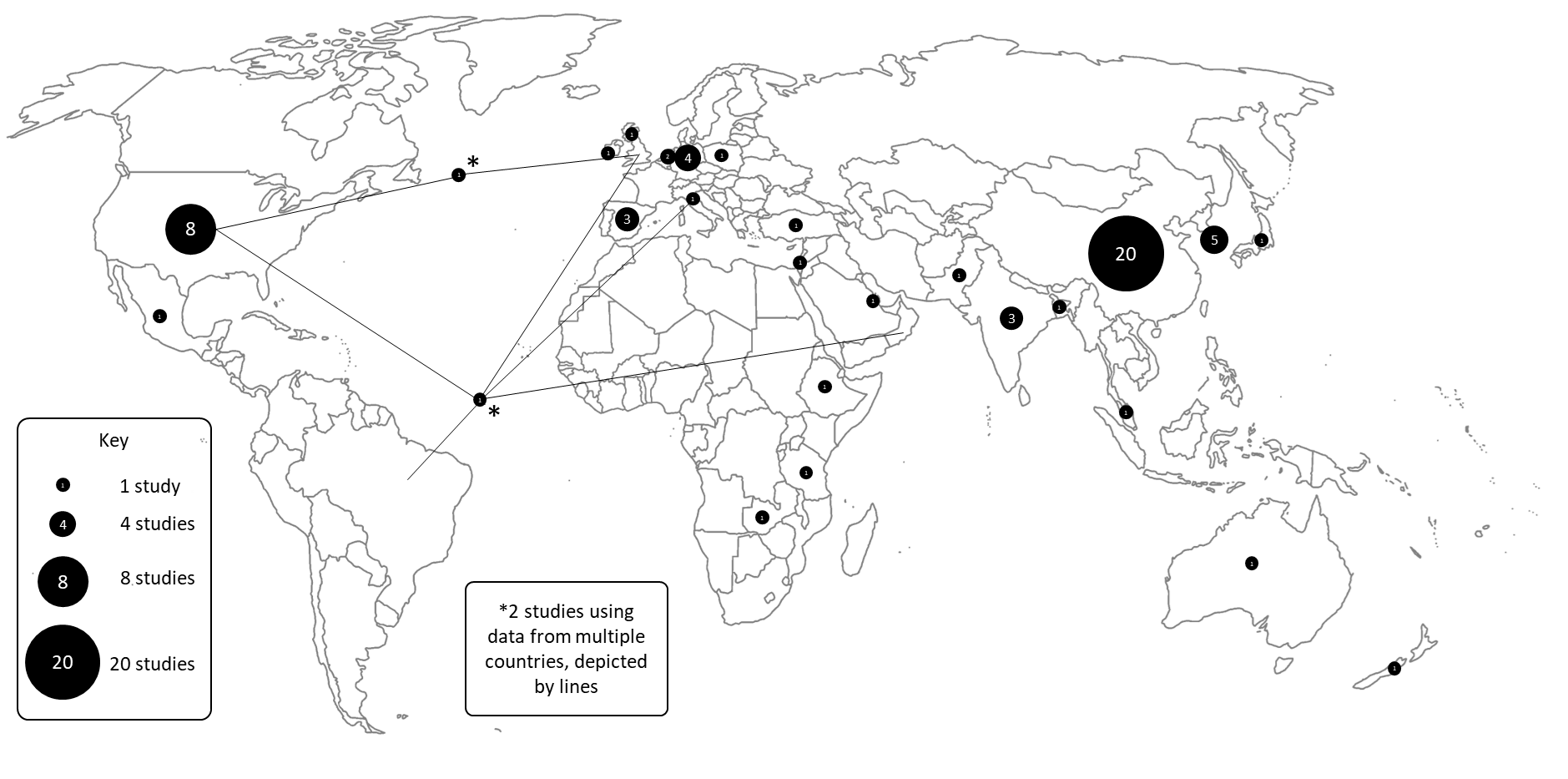


**Figure S2:**

**
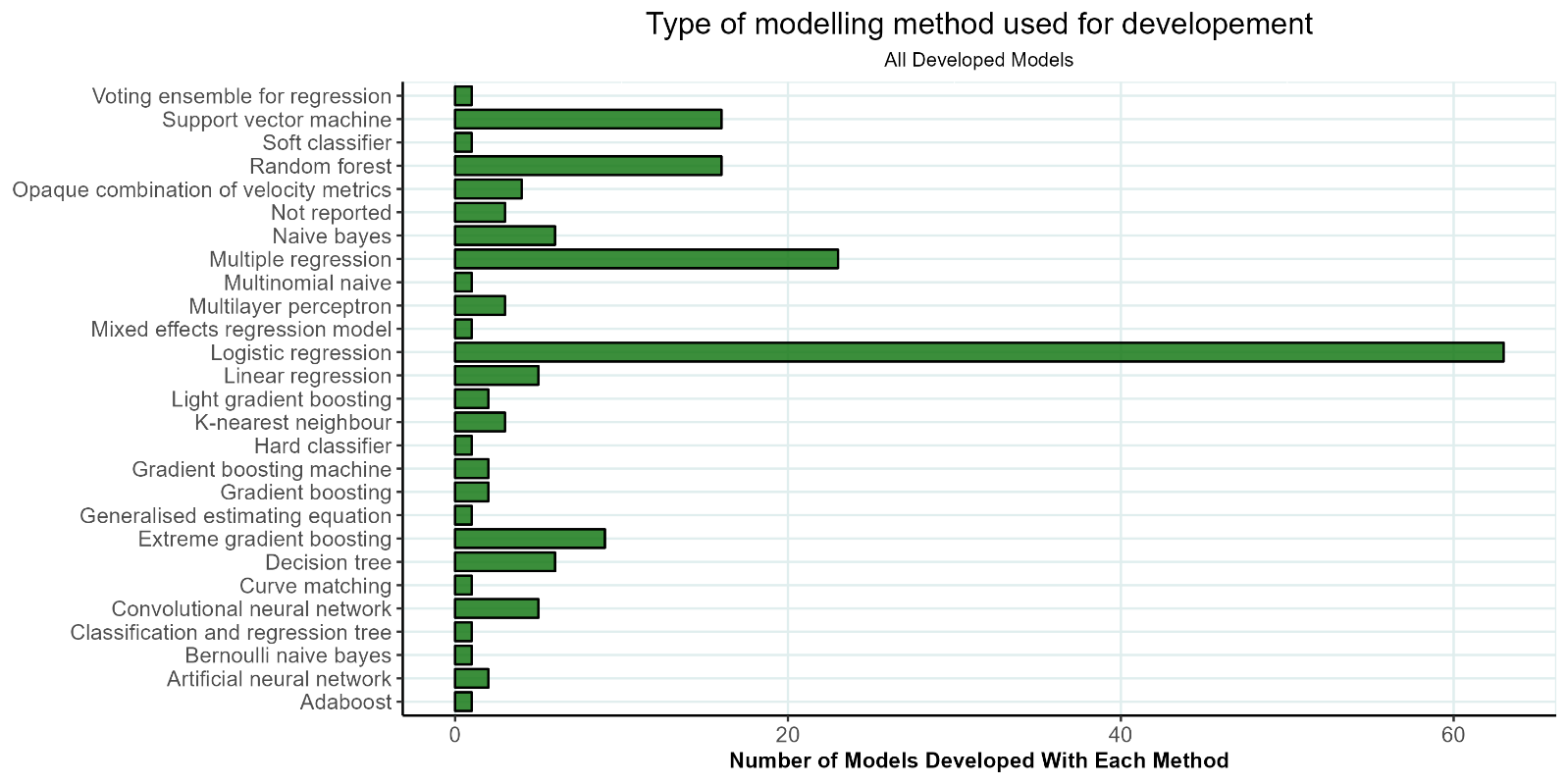
**

**Table S6:**

Methods employed for all models developed reported by type

| **Model Method** | **n, %** |
| --- | --- |
| ***Regression Based Method (n=97)*** | |
| Logistic regression | 63, 35.0% |
| Multiple linear regression | 23, 12.8% |
| Linear regression | 5, 2.7% |
| Opaque combination of velocity metrics (likely regression) | 4, 2.2% |
| Mixed effects regression model | 1, 0.6% |
| Generalised estimating equation | 1, 0.6% |
| ***Flexible Machine Learning Method (n=45)*** | |
| Support vector machine | 16, 8.9% |
| Naive bayes | 6, 3.3% |
| Decision tree | 6, 3.3% |
| Convolutional neural network | 5, 2.7% |
| Multilayer perceptron | 3, 1.6% |
| K-nearest neighbour | 3, 1.6% |
| Artificial neural network | 2, 1.1% |
| Multinomial naïve | 1, 0.6% |
| Curve matching | 1, 0.6% |
| Classification and regression tree | 1, 0.6% |
| Bernoulli naive bayes | 1, 0.6% |
| ***Ensemble Machine Learning Method (n=35)*** | |
| Random forest | 16, 8.9% |
| Extreme gradient boosting | 9, 5.0% |
| Gradient boosting | 4, 2.2% |
| Light gradient boosting | 2, 1.1% |
| Voting ensemble for regression | 1, 0.6% |
| Soft classifier | 1, 0.6% |
| Hard classifier | 1, 0.6% |
| Adaboost | 1, 0.6% |
| ***Model development method not reported*** | |
| Not reported | 3, 1.6% |

**Figure S3:**

**
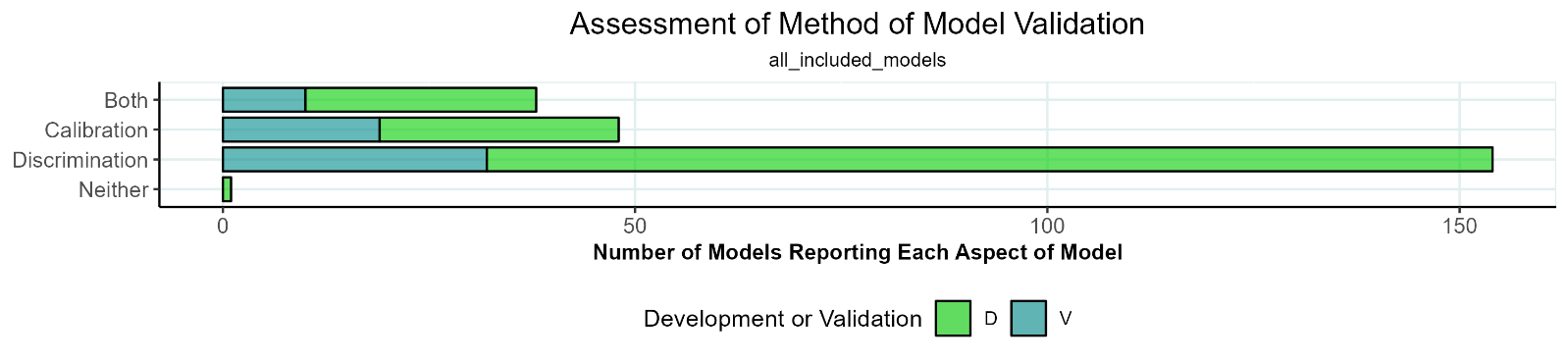
**

Method of analysis of accuracy of predictions from models reviewed

**Table S7:**

Individual question ratings for all developed and validated models

|  |  | **Developed models (n=180)**  **n, %** | | | | |  | **Validated models (n=61)**  **n, %** | | | | |
| --- | --- | --- | --- | --- | --- | --- | --- | --- | --- | --- | --- | --- |
|  | **Question** | **Y** | **PY** | **PN** | **N** | **NI** |  | **Y** | **PY** | **PN** | **N** | **NI** |
| 1.1 | Were appropriate data sources used, e.g., cohort, randomized controlled trial, or nested case control study data? | n = 78,  43% | n = 5,  3% | n = 65,  36% | n = 14,  8% | n = 18,  10% |  | n = 40,  66% | n = 3,  5% | n = 3,  5% | n = 15,  24% | n = 0,  0% |
| 1.2 | Were all inclusions and exclusions of participants appropriate? | n = 30,  17% | n = 32,  18% | n = 28,  15% | n = 63,  35% | n = 27,  15% |  | n = 21,  35% | n = 10,  16% | n = 0,  0% | n = 27,  44% | n = 3,  5% |
|  |  |  |  |  |  |  |  |  |  |  |  |  |
| 2.1 | Were predictors defined and assessed in a similar way for all participants? | n = 39,  21% | n = 13,  7% | n = 55,  31% | n = 55,  31% | n = 18,  10% |  | n = 26,  42% | n = 11,  18% | n = 1,  2% | n = 20,  33% | n = 3,  5% |
| 2.2 | Were predictor assessments made without knowledge of outcome data? | n = 91,  51% | n = 32,  18% | n = 29,  16% | n = 13,  7% | n = 15,  8% |  | n = 52,  85% | n = 0,  0% | n = 0,  0% | n = 2,  3% | n = 7,  12% |
| 2.3 | Are all predictors available at the time the model is intended to be used? | n = 168,  93% | n = 0,  0% | n = 0,  0% | n = 10,  6% | n = 2,  1% |  | n = 61,  100% | n = 0,  0% | n = 0,  0% | n = 0,  0% | n = 0,  0% |
|  |  |  |  |  |  |  |  |  |  |  |  |  |
| 3.1 | Was the outcome determined appropriately? | n = 35,  19% | n = 57,  32% | n = 27,  15% | n = 50,  28% | n = 11,  6% |  | n = 24,  39% | n = 30,  49% | n = 3,  5% | n = 3,  5% | n = 1,  2% |
| 3.2 | Was a prespecified or standard outcome definition used? | n = 157,  87% | n = 12,  7% | n = 0,  0% | n = 11,  6% | n = 0,  0% |  | n = 54,  88% | n = 3,  5% | n = 0,  0% | n = 3,  5% | n = 1,  2% |
| 3.3 | Were predictors excluded from the outcome definition? | n = 168  93% | n = 0,  0% | n = 0,  0% | n = 12,  7% | n = 0,  0% |  | n = 55,  90% | n = 3,  5% | n = 0,  0% | n = 3,  5% | n = 0,  0% |
| 3.4 | Was the outcome defined and determined in a similar way for all participants? | n = 19,  10% | n = 92,  52% | n = 21,  12% | n = 29,  16% | n = 19,  10% |  | n = 19,  31% | n = 34,  56% | n = 1,  2% | n = 7,  11% | n = 0,  0% |
| 3.5 | Was the outcome determined without knowledge of predictor information? | n = 0,  0% | n = 76,  42% | n = 17,  10% | n = 31,  17% | n = 56,  31% |  | n = 0,  0% | n = 38,  63% | n = 2,  3% | n = 2,  3% | n = 19,  31% |
| 3.6 | Was the time interval between predictor assessment and outcome determination appropriate? | n = 88,  49% | n = 3,  2% | n = 19,  10% | n = 52,  29% | n = 18,  10% |  | n = 43,  71% | n = 6,  10% | n = 3,  5% | n = 7,  11% | n = 2,  3% |
|  |  |  |  |  |  |  |  |  |  |  |  |  |
|  |  | **Developed Models** | | | | |  | **Validated Models** | | | | |
|  | **Question** | **Y** | **PY** | **PN** | **N** | **NI** |  | **Y** | **PY** | **PN** | **N** | **NI** |
| 4.1 | Were there a reasonable number of participants with the outcome? | n = 29,  16% | n = 58,  32% | n = 13,  8% | n = 49,  27% | n = 31,  17% |  | n = 21,  34% | n = 21,  34% | n = 3,  5% | n = 16,  27% | n = 0,  0% |
| 4.2 | Were continuous and categorical predictors handled appropriately? | n = 24,  13% | n = 14,  8% | n = 1,  1% | n = 121,  67% | n = 20,  11% |  | n = 53,  87% | n = 0,  0% | n = 0,  0% | n = 7,  11% | n = 1,  2% |
| 4.3 | Were all enrolled participants included in the analysis? | n = 0,  0% | n = 0,  0% | n = 1,  1% | n = 143,  79% | n = 36,  20% |  | n = 7,  11% | n = 0,  0% | n = 0,  0% | n = 37,  61% | n = 17,  28% |
| 4.4 | Were participants with missing data handled appropriately? | n = 11,  6% | n = 2,  1% | n = 19,  11% | n = 114,  63% | n = 34,  19% |  | n = 0,  0% | n = 2,  3% | n = 0,  0% | n = 38,  62% | n = 21,  35% |
| 4.5 | Was selection of predictors based on univariable analysis avoided? | n = 88,  49% | n = 3,  2% | n = 12,  6% | n = 50,  28% | n = 27,  15% |  |  |  |  |  |  |
| 4.6 | Were complexities in the data (e.g. censoring, competing risks, sampling of control participants) accounted for appropriately? | n = 38,  21% | n = 0,  0% | n = 2,  1% | n = 115,  64% | n = 25,  14% |  | n = 8,  13% | n = 0,  0% | n = 2,  3% | n = 45,  74% | n = 6,  10% |
| 4.7 | Were relevant model performance measures evaluated appropriately? | n = 8,  4% | n = 13,  7% | n = 3,  2% | n = 156,  87% | n = 0,  0% |  | n = 4,  6% | n = 0,  0% | n = 3,  5% | n = 54,  89% | n = 0,  0% |
| 4.8 | Were model overfitting and optimism in model performance accounted for? | n = 16,  9% | n = 11,  6% | n = 11,  6% | n = 103,  57% | n = 39,  22% |  |  |  |  |  |  |
| 4.9 | Do predictors and their assigned weights in the final model correspond to the results from the reported multivariable analysis? | n = 29,  16% | n = 2,  1% | n = 3,  2% | n = 14,  8% | n = 132,  73% |  |  |  |  |  |  |

**Figure S4:**

Heat map of individual question ratings. Each model on separate row.

| **Key: Colour of question responses** | | | | |  |
| --- | --- | --- | --- | --- | --- |
| **No:** | **Probably no:** | **No information** | **Probably Yes:** | **Yes:** | **Not applicable:** |
| N | PN | NI | PY | Y |  |

|  | **Domain 1: Participants** | | **Domain 2:  Predictors** | | | **Domain 3:  Outcome** | | | | | | **Domain 4:  Analysis** | | | | | | | | |
| --- | --- | --- | --- | --- | --- | --- | --- | --- | --- | --- | --- | --- | --- | --- | --- | --- | --- | --- | --- | --- |
| **Article Code:** | **Q  1.1** | **Q  1.2** | **Q  2.1** | **Q  2.2** | **Q  2.3** | **Q  3.1** | **Q  3.2** | **Q  3.3** | **Q  3.4** | **Q  3.5** | **Q  3.6** | **Q  4.1** | **Q  4.2** | **Q  4.3** | **Q  4.4** | **Q  4.5** | **Q  4.6** | **Q  4.7** | **Q  4.8** | **Q  4.9** |
| A003 | Y | N | N | Y | Y | PY | Y | Y | PY | PY | Y | N | N | N | N | N | Y | Y | Y | Y |
| A003 | Y | N | N | Y | Y | PY | Y | Y | PY | PY | Y | N | N | N | N | N | Y | Y | Y | Y |
| A004 | Y | PY | Y | Y | Y | Y | Y | Y | Y | PN | Y | PY | PY | N | PN | Y | Y | N | NI | N |
| A004 | Y | PY | Y | Y | Y | Y | Y | Y | Y | PN | Y | PY | PY | N | PN | Y | Y | N | NI | N |
| A004 | Y | PY | Y | Y | Y | Y | Y | Y | Y | PN | Y | PY | PY | N | PN | Y | Y | N | NI | N |
| A004 | Y | PY | Y | Y | Y | Y | Y | Y | Y | PN | Y | PY | PY | N | PN | Y | Y | N | NI | N |
| A004 | Y | PY | Y | Y | Y | Y | Y | Y | Y | PN | Y | PY | PY | N | PN | Y | Y | N | NI | N |
| A004 | Y | PY | Y | Y | Y | Y | Y | Y | Y | PN | Y | PY | PY | N | PN | Y | Y | N | NI | N |
| A004 | Y | PY | Y | Y | Y | Y | Y | Y | Y | PN | Y | PY | PY | N | PN | Y | Y | N | NI | N |
| A004 | Y | PY | Y | Y | Y | Y | Y | Y | Y | PN | Y | PY | PY | N | PN | Y | Y | N | NI | N |
| A005 | Y | N | PY | Y | Y | PY | Y | N | PY | PN | Y | PY | Y | N | PY | Y | NI | N | NI | NI |
| A006 | Y | PY | Y | N | Y | N | Y | Y | N | N | Y | N | N | N | N | Y | N | N | PN | NI |
| A006 | Y | PY | Y | N | Y | N | Y | Y | N | N | Y | N | N | N | N | Y | N | N | PN | NI |
| A006 | Y | PY | Y | N | Y | N | Y | Y | N | N | Y | N | N | N | N | Y | N | N | PN | NI |
| A006 | Y | PY | Y | N | Y | N | Y | Y | N | N | Y | N | N | N | N | Y | N | N | PN | NI |
| A006 | Y | PY | Y | N | Y | N | Y | Y | N | N | Y | N | N | N | N | Y | N | N | PN | NI |
| A006 | Y | PY | Y | N | Y | N | Y | Y | N | N | Y | N | N | N | N | Y | N | N | PN | NI |
| A006 | Y | PY | Y | N | Y | N | Y | Y | N | N | Y | N | N | N | N | Y | N | N | PN | NI |
| A006 | Y | PY | Y | N | Y | N | Y | Y | N | N | Y | N | N | N | N | Y | N | N | PN | NI |
| A007 | PN | NI | N | Y | Y | N | N | Y | PN | NI | NI | PY | NI | NI | NI | Y | NI | N | NI | NI |
| A007 | N | NI | PN | Y | Y | N | N | N | PN | NI | N | N | NI | N | NI |  | NI | N |  |  |
| A008 | Y | Y | Y | Y | Y | Y | Y | Y | PY | NI | Y | Y | N | N | N | N | Y | Y | NI | PN |
| A008 | Y | Y | Y | Y | Y | Y | Y | Y | PY | NI | Y | Y | N | N | N | N | Y | Y | NI | PN |
| A008 | Y | Y | Y | Y | Y | Y | Y | Y | PY | NI | Y | Y | N | N | N | N | Y | Y | NI | PN |
| A010 | Y | Y | Y | Y | Y | Y | Y | N | PY | NI | Y | PN | Y | N | N | Y | NI | PY | NI | NI |
| A010 | Y | Y | Y | Y | Y | Y | Y | N | PY | NI | Y | PN | Y | N | N | Y | NI | PY | NI | NI |
| A010 | Y | Y | Y | Y | Y | Y | Y | N | PY | NI | Y | PN | Y | N | N | Y | NI | PY | NI | NI |
| A012 | N | N | Y | NI | Y | Y | Y | Y | Y | NI | Y | PY | Y | N | N |  | N | N |  |  |
| A012 | N | N | Y | NI | Y | Y | Y | Y | Y | NI | Y | PY | Y | N | N |  | N | N |  |  |
| A012 | N | N | Y | NI | Y | Y | Y | Y | Y | NI | Y | PY | Y | N | N |  | N | N |  |  |
| A012 | N | N | Y | NI | Y | Y | Y | Y | Y | NI | Y | PY | Y | N | N |  | N | N |  |  |
| A012 | N | N | Y | NI | Y | Y | Y | Y | Y | NI | Y | PY | Y | N | N |  | N | N |  |  |
| A013 | Y | PY | Y | Y | Y | Y | Y | Y | PY | NI | Y | N | N | NI | NI | PN | NI | Y | NI | Y |
| A013 | Y | PY | Y | Y | Y | Y | Y | Y | PY | NI | Y | N | N | NI | NI |  | NI | Y |  |  |
| A013 | Y | PY | Y | Y | Y | Y | Y | Y | PY | NI | Y | N | N | NI | NI |  | NI | Y |  |  |
| A014 | Y | N | N | Y | Y | Y | Y | Y | PY | NI | Y | PY | N | N | N | N | N | Y | N | PY |
| A015 | NI | NI | NI | NI | N | NI | Y | Y | NI | NI | N | N | NI | NI | NI | NI | NI | N | NI | NI |
| A015 | NI | NI | NI | NI | N | NI | Y | Y | NI | NI | N | N | NI | NI | NI | NI | NI | N | NI | NI |
| A015 | NI | NI | NI | NI | N | NI | Y | Y | NI | NI | N | N | NI | NI | NI | NI | NI | N | NI | NI |
| A015 | NI | NI | NI | NI | N | NI | Y | Y | NI | NI | N | N | NI | NI | NI | NI | NI | N | NI | NI |
| A015 | NI | NI | NI | NI | N | NI | Y | Y | NI | NI | N | N | NI | NI | NI | NI | NI | N | NI | NI |
| A015 | NI | NI | NI | NI | N | NI | Y | Y | NI | NI | N | N | NI | NI | NI | NI | NI | N | NI | NI |
| A015 | NI | NI | NI | NI | N | NI | Y | Y | NI | NI | N | N | NI | NI | NI | NI | NI | N | NI | NI |
| A015 | NI | NI | NI | NI | N | NI | Y | Y | NI | NI | N | N | NI | NI | NI | NI | NI | N | NI | NI |
| A015 | NI | NI | NI | NI | N | NI | Y | Y | NI | NI | N | N | NI | NI | NI | NI | NI | N | NI | NI |
| A015 | NI | NI | NI | NI | N | NI | Y | Y | NI | NI | N | N | NI | NI | NI | NI | NI | N | NI | NI |
| A016 | Y | N | PN | Y | Y | PN | Y | Y | NI | PY | Y | N | N | N | N | Y | N | N | NI | N |
| A020 | Y | Y | Y | N | Y | N | N | Y | N | N | N | Y | N | N | N | N | N | PN | N | NI |
| A022 | Y | Y | Y | Y | Y | Y | N | Y | Y | PY | Y | Y | Y | N | NI | Y | N | N | N | Y |
| A022 | Y | NI | PY | Y | Y | NI | NI | Y | PY | PY | Y | PY | Y | NI | NI |  | NI | N |  |  |
| A022 | Y | Y | N | Y | Y | Y | Y | Y | Y | PY | Y | Y | Y | NI | NI |  | Y | N |  |  |
| A022 | Y | Y | N | Y | Y | Y | Y | Y | Y | PY | Y | Y | Y | NI | NI |  | Y | N |  |  |
| A022 | Y | Y | N | Y | Y | Y | Y | Y | Y | PY | Y | Y | Y | NI | NI |  | Y | N |  |  |
| A023 | Y | NI | Y | Y | Y | Y | Y | Y | PY | PY | Y | PN | PY | PN | NI | PN | PN | N | NI | NI |
| A025 | Y | Y | PY | PY | Y | PY | Y | Y | PY | PN | Y | Y | Y | N | N | Y | N | N | PN | NI |
| A026 | N | Y | Y | N | Y | NI | Y | Y | PN | N | NI | Y | N | N | N | Y | PN | Y | Y | PY |
| A027 | Y | NI | Y | Y | Y | PY | Y | Y | Y | PY | Y | PY | NI | NI | NI | NI | N | N | PN | NI |
| A028 | Y | Y | PY | Y | Y | Y | Y | Y | PY | PY | Y | PN | N | NI | NI | PN | N | N | NI | NI |
| A028 | Y | Y | PY | Y | Y | Y | Y | Y | PY | PY | Y | PN | N | NI | NI | PN | N | N | NI | NI |
| A028 | Y | Y | PY | Y | Y | Y | Y | Y | PY | PY | Y | PN | N | NI | NI | PN | N | N | NI | NI |
| A029 | PY | N | Y | Y | Y | PY | Y | Y | PY | PY | Y | Y | Y | N | N |  | N | N |  |  |
| A031 | PY | N | PY | Y | Y | Y | Y | Y | PY | PY | NI | Y | Y | NI | NI |  | N | N |  |  |
| A031 | PY | N | PY | Y | Y | Y | Y | Y | PY | PY | NI | Y | Y | NI | NI |  | N | N |  |  |
| A032 | N | N | Y | N | Y | N | PY | Y | PN | NI | N | PY | N | N | N | N | N | N | NI | NI |
| A032 | N | N | Y | N | Y | N | PY | Y | PN | NI | N | PY | N | N | N | N | N | N | NI | NI |
| A035 | Y | N | PN | NI | Y | PY | Y | Y | PY | NI | NI | N | N | N | N | NI | N | N | NI | N |
| A035 | Y | N | PN | NI | Y | PY | Y | Y | PY | NI | NI | N | N | N | N | NI | N | N | NI | N |
| A035 | Y | N | PN | NI | Y | PY | Y | Y | PY | NI | NI | N | N | N | N | NI | N | N | NI | N |
| A036 | Y | Y | N | Y | Y | Y | Y | Y | N | PY | Y | Y | N | N | N | PN | N | N | N | Y |
| A036 | Y | Y | N | Y | Y | Y | Y | Y | N | PY | Y | Y | N | N | N | PN | N | N | N | Y |
| A036 | Y | Y | N | Y | Y | Y | Y | Y | N | PY | Y | Y | N | N | N | PN | N | N | N | Y |
| A036 | Y | Y | N | Y | Y | Y | Y | Y | N | PY | Y | Y | N | N | N | PN | N | N | N | Y |
| A036 | Y | Y | Y | Y | Y | Y | Y | Y | N | PY | Y | Y | Y | N | N |  | N | N |  |  |
| A036 | Y | Y | Y | Y | Y | Y | Y | Y | N | PY | Y | Y | Y | N | N |  | N | N |  |  |
| A037 | Y | N | Y | Y | Y | Y | Y | Y | Y | NI | Y | PY | Y | NI | NI |  | N | N |  |  |
| A037 | Y | N | Y | Y | Y | Y | Y | Y | Y | NI | Y | PY | Y | NI | NI |  | N | N |  |  |
| A038 | Y | Y | Y | Y | Y | PY | Y | Y | PY | NI | Y | PY | Y | N | N |  | N | N |  |  |
| A039 | Y | PY | PY | Y | Y | PY | Y | Y | PY | NI | Y | Y | Y | N | N | NI | NI | N | N | NI |
| A039 | Y | PY | Y | Y | Y | PY | Y | Y | PY | PY | Y | Y | Y | N | N |  | NI | Y |  |  |
| A040 | Y | Y | N | Y | Y | PY | Y | Y | PY | PN | PN | PY | N | N | NI | PN | N | N | N | NI |
| A040 | Y | Y | N | Y | Y | PY | Y | Y | PY | PN | PN | PY | N | N | NI | PN | N | N | N | NI |
| A040 | Y | Y | N | Y | Y | PY | Y | Y | PY | PN | PN | PY | N | N | NI | PN | N | N | N | NI |
| A041 | Y | Y | PY | Y | Y | PY | Y | Y | PY | NI | Y | N | N | N | N | N | N | N | N | N |
| A043 | PN | PY | N | Y | Y | PN | PY | Y | N | NI | PN | PY | N | N | N | NI | N | PY | N | NI |
| A043 | PN | PY | N | Y | Y | PN | PY | Y | N | NI | PN | PY | N | N | N | NI | N | PY | N | NI |
| A043 | PN | PY | N | Y | Y | PN | PY | Y | N | NI | PN | PY | N | N | N | NI | N | PY | N | NI |
| A043 | PN | PY | N | Y | Y | PN | PY | Y | N | NI | PN | PY | N | N | N | NI | N | PY | N | NI |
| A043 | PN | PY | N | Y | Y | PN | PY | Y | N | NI | PN | PY | N | N | N | NI | N | PY | N | NI |
| A043 | PN | PY | N | Y | Y | PN | PY | Y | N | NI | PN | PY | N | N | N | NI | N | PY | N | NI |
| A043 | PN | PY | N | Y | Y | PN | PY | Y | N | NI | PN | PY | N | N | N | NI | N | PY | N | NI |
| A043 | PN | PY | N | Y | Y | PN | PY | Y | N | NI | PN | PY | N | N | N | NI | N | PY | N | NI |
| A043 | PN | PY | N | Y | Y | PN | PY | Y | N | NI | PN | PY | N | N | N | NI | N | PY | N | NI |
| A043 | PN | PY | N | Y | Y | PN | PY | Y | N | NI | PN | PY | N | N | N | NI | N | PY | N | NI |
| A043 | PN | PY | N | Y | Y | PN | PY | Y | N | NI | PN | N | Y | N | N |  | N | PN |  |  |
| A043 | PN | PY | N | Y | Y | PN | PY | Y | N | NI | PN | N | Y | N | N |  | N | PN |  |  |
| A043 | PN | PY | N | Y | Y | PN | PY | Y | N | NI | PN | N | Y | N | N |  | N | PN |  |  |
| A045 | Y | Y | PY | Y | Y | N | Y | Y | PY | NI | Y | N | N | NI | NI | N | N | N | NI | Y |
| A045 | Y | Y | PY | Y | Y | N | Y | Y | PY | NI | Y | N | N | NI | NI | N | N | N | NI | Y |
| A046 | Y | N | Y | Y | Y | PY | Y | Y | Y | PY | Y | PY | Y | NI | NI |  | NI | N |  |  |
| A049 | NI | NI | NI | Y | Y | PY | Y | Y | NI | NI | Y | N | NI | NI | NI | N | NI | N | N | NI |
| A049 | NI | NI | NI | Y | Y | PY | Y | Y | NI | NI | Y | N | NI | NI | NI | N | NI | N | N | NI |
| A049 | NI | NI | NI | Y | Y | PY | Y | Y | NI | NI | Y | N | NI | NI | NI | N | NI | N | N | NI |
| A049 | NI | NI | NI | Y | Y | PY | Y | Y | NI | NI | Y | N | NI | NI | NI | N | NI | N | N | NI |
| A049 | NI | NI | NI | Y | Y | PY | Y | Y | NI | NI | Y | N | N | NI | NI | N | NI | N | N | Y |
| A049 | NI | NI | NI | Y | Y | PY | Y | Y | NI | NI | Y | N | N | NI | NI | N | NI | N | N | Y |
| A049 | NI | NI | NI | Y | Y | PY | Y | Y | NI | NI | Y | N | N | NI | NI | N | NI | N | N | Y |
| A049 | NI | NI | NI | Y | Y | PY | Y | Y | NI | NI | Y | N | N | NI | NI | N | NI | N | N | Y |
| A050 | PY | NI | PN | PN | Y | PN | Y | Y | PN | N | N | Y | N | NI | N | Y | Y | N | Y | NI |
| A050 | PY | NI | PN | PN | Y | PN | Y | Y | PN | N | N | Y | N | NI | N | Y | Y | N | Y | NI |
| A050 | PY | NI | PN | PN | Y | PN | Y | Y | PN | N | N | Y | N | NI | N | Y | Y | N | Y | NI |
| A050 | PY | NI | PN | PN | Y | PN | Y | Y | PN | N | N | Y | N | NI | N | Y | Y | N | Y | NI |
| A050 | PY | NI | PN | PN | Y | PN | Y | Y | PN | N | N | Y | N | NI | N | Y | Y | N | Y | NI |
| A051 | Y | N | Y | NI | Y | Y | Y | Y | Y | NI | Y | Y | N | N | NI | PY | Y | N | N | N |
| A052 | N | Y | N | Y | Y | PY | Y | Y | PY | PY | Y | Y | Y | Y | N |  | N | N |  |  |
| A052 | N | Y | N | Y | Y | PY | Y | Y | PY | PY | Y | Y | Y | Y | N |  | N | N |  |  |
| A052 | N | Y | N | Y | Y | PY | Y | Y | PY | PY | Y | Y | Y | Y | N |  | N | N |  |  |
| A052 | N | Y | N | Y | Y | PY | Y | Y | PY | PY | Y | Y | Y | Y | N |  | N | N |  |  |
| A052 | N | Y | N | Y | Y | PY | Y | Y | PY | PY | Y | Y | Y | Y | N |  | N | N |  |  |
| A052 | N | Y | N | Y | Y | PY | Y | Y | PY | PY | Y | Y | Y | Y | N |  | N | N |  |  |
| A052 | N | Y | N | Y | Y | PY | Y | Y | PY | PY | Y | Y | Y | Y | N |  | N | N |  |  |
| A053 | Y | NI | Y | Y | Y | PY | Y | Y | PY | NI | N | PY | Y | NI | NI |  | N | N |  |  |
| A054 | Y | PY | PY | Y | Y | PY | Y | Y | PY | PY | Y | N | Y | N | N |  | N | Y |  |  |
| A058 | N | NI | N | N | NI | N | Y | Y | PN | N | Y | Y | N | N | N | N | N | N | N | Y |
| A059 | N | PN | Y | Y | Y | Y | Y | Y | Y | NI | PN | PN | N | N | N | N | N | N | N | Y |
| A059 | N | PN | Y | Y | Y | Y | Y | Y | Y | NI | PN | PN | N | N | N | N | N | N | N | Y |
| A059 | N | PN | Y | Y | Y | Y | Y | Y | Y | NI | PN | PN | N | N | N | N | N | N | N | Y |
| A059 | N | PN | Y | Y | Y | Y | Y | Y | Y | NI | PN | PY | N | N | N | N | N | N | N | Y |
| A059 | N | PN | Y | Y | Y | Y | Y | Y | Y | NI | PN | PY | N | N | N | N | N | N | N | Y |
| A059 | N | PN | Y | Y | Y | Y | Y | Y | Y | NI | PN | PY | N | N | N | N | N | N | N | Y |
| A063 | Y | Y | N | Y | Y | PY | Y | Y | PY | NI | PY | Y | NI | NI | N | Y | N | N | N | NI |
| A063 | Y | Y | N | Y | Y | PY | Y | Y | PY | NI | PY | Y | NI | NI | N | Y | N | N | N | NI |
| A063 | Y | Y | N | Y | Y | PY | Y | Y | PY | NI | PY | Y | NI | NI | N | Y | N | N | N | NI |
| A063 | Y | Y | PY | Y | Y | PY | Y | Y | PY | NI | Y | Y | Y | N | NI |  | N | N |  |  |
| A064 | PN | N | PN | PN | Y | Y | Y | Y | N | PN | Y | Y | NI | N | N | NI | N | N | N | NI |
| A070 | Y | PY | Y | Y | Y | PY | Y | Y | PY | NI | PY | N | Y | N | N |  | Y | N |  |  |
| A070 | Y | PY | Y | Y | Y | PY | Y | Y | PY | NI | PY | N | Y | N | N |  | Y | N |  |  |
| A070 | Y | PY | Y | Y | Y | PY | Y | Y | PY | NI | PY | N | Y | N | N |  | Y | N |  |  |
| A078 | Y | N | N | Y | Y | PY | Y | Y | PY | PY | Y | PY | N | N | N | PY | N | N | N | NI |
| A078 | Y | N | N | Y | Y | PY | Y | Y | PY | PY | Y | PY | N | N | N | PY | N | N | N | NI |
| A078 | Y | N | N | Y | Y | PY | Y | Y | PY | PY | Y | PY | Y | N | N |  | PN | N |  |  |
| A078 | Y | N | N | Y | Y | PY | Y | Y | PY | PY | Y | PY | Y | N | N |  | PN | N |  |  |
| A081 | Y | N | NI | Y | Y | PY | Y | Y | PY | PY | Y | N | N | N | NI |  | N | N |  |  |
| A081 | Y | N | NI | Y | Y | PY | Y | Y | PY | PY | Y | N | N | N | NI |  | N | N |  |  |
| A081 | Y | N | NI | Y | Y | PY | Y | Y | PY | PY | Y | N | N | N | NI |  | N | N |  |  |
| A082 | Y | Y | N | Y | Y | PY | Y | Y | PY | PY | Y | Y | N | N | N | Y | N | N | N | NI |
| A082 | Y | Y | N | Y | Y | PY | Y | Y | PY | PY | Y | Y | N | N | N | Y | N | N | N | NI |
| A082 | Y | Y | N | Y | Y | PY | Y | Y | PY | PY | Y | Y | N | N | N | Y | N | N | N | NI |
| A082 | Y | Y | N | Y | Y | PY | Y | Y | PY | PY | Y | Y | N | N | N | Y | N | N | N | NI |
| A082 | Y | Y | Y | Y | Y | PY | Y | Y | PY | PY | Y | Y | Y | N | N |  | N | N |  |  |
| A086 | Y | N | PY | Y | Y | PY | Y | Y | PY | PY | Y | PN | Y | NI | NI |  | N | N |  |  |
| A086 | Y | N | PY | Y | Y | PY | Y | Y | PY | PY | Y | PN | Y | NI | NI |  | N | N |  |  |
| A086 | Y | N | PY | Y | Y | PY | Y | Y | PY | PY | Y | PN | Y | NI | NI |  | N | N |  |  |
| A091 | Y | N | Y | Y | Y | PY | Y | Y | PY | PY | Y | Y | Y | N | NI |  | N | N |  |  |
| A099 | Y | N | Y | NI | Y | Y | Y | Y | Y | PN | Y | Y | Y | NI | PY | NI | Y | N | N | Y |
| A099 | Y | N | Y | NI | Y | Y | Y | Y | Y | PN | Y | Y | Y | NI | PY |  | Y | N |  |  |
| A099 | Y | N | Y | NI | Y | Y | Y | Y | Y | PN | Y | Y | Y | NI | PY |  | Y | N |  |  |
| A108 | Y | PY | N | Y | Y | PY | Y | Y | PY | PY | Y | N | Y | N | N | N | Y | N | Y | NI |
| A108 | Y | PY | N | Y | Y | PY | Y | Y | PY | PY | Y | N | Y | N | N | N | Y | N | Y | NI |
| A108 | Y | PY | N | Y | Y | PY | Y | Y | PY | PY | Y | N | Y | N | N | N | Y | N | Y | NI |
| A109 | PN | PN | PN | PN | Y | PN | Y | Y | PN | N | Y | PY | N | N | Y | N | Y | N | PY | NI |
| A109 | PN | PN | PN | PN | Y | PN | Y | Y | PN | N | Y | PY | N | N | Y | N | Y | N | PY | NI |
| A109 | PN | PN | PN | PN | Y | PN | Y | Y | PN | N | Y | PY | N | N | Y | N | Y | N | PY | NI |
| A109 | PN | PN | PN | PN | Y | PN | Y | Y | PN | N | Y | PY | N | N | Y | N | Y | N | PY | NI |
| A109 | PN | PN | PN | PN | Y | PN | Y | Y | PN | N | Y | PY | N | N | Y | N | Y | N | PY | NI |
| A109 | PN | PN | PN | PN | Y | PN | Y | Y | PN | N | Y | PY | N | N | Y | N | Y | N | PY | NI |
| A109 | PN | PN | PN | PN | Y | PN | Y | Y | PN | N | Y | PY | N | N | Y | N | Y | N | PY | NI |
| A109 | PN | PN | PN | PN | Y | PN | Y | Y | PN | N | Y | PY | N | N | Y | N | Y | N | PY | NI |
| A109 | PN | PN | PN | PN | Y | PN | Y | Y | PN | N | Y | PY | N | N | Y | N | Y | N | PY | NI |
| A109 | PN | PN | PN | PN | Y | PN | Y | Y | PN | N | Y | PY | N | N | Y | N | Y | N | PY | NI |
| A109 | PN | PN | PN | PN | Y | PN | Y | Y | PN | N | Y | PY | N | N | Y | N | Y | N | PY | NI |
| A117 | Y | N | PN | PN | Y | N | Y | Y | N | N | NI | Y | Y | N | NI | Y | Y | N | N | Y |
| A118 | Y | N | PN | Y | Y | PY | Y | Y | PY | PN | Y | PN | N | N | N | N | Y | PN | Y | Y |
| A118 | Y | N | PN | Y | Y | PY | Y | Y | PY | PN | Y | PN | N | N | N | N | Y | PN | Y | Y |
| A120 | PN | PN | N | PN | Y | PY | Y | Y | PY | PY | NI | PY | Y | N | PN | Y | N | N | N | Y |
| A120 | PN | PN | N | PN | Y | PY | Y | Y | PY | PY | NI | PY | Y | N | PN | Y | N | N | N | NI |
| A120 | PN | PN | N | PN | Y | PY | Y | Y | PY | PY | NI | PY | Y | N | PN | Y | N | N | N | NI |
| A120 | PN | PN | N | PN | Y | PY | Y | Y | PY | PY | NI | PY | Y | N | PN | Y | N | N | N | NI |
| A120 | PN | PN | N | PN | Y | PY | Y | Y | PY | PY | NI | PY | Y | N | PN | Y | N | N | N | NI |
| A120 | PN | PN | N | PN | Y | PY | Y | Y | PY | PY | NI | PY | Y | N | PN | Y | N | N | N | NI |
| A120 | PN | PN | N | PN | Y | PY | Y | Y | PY | PY | NI | PY | Y | N | PN | Y | N | N | N | NI |
| A120 | PN | PN | N | PN | Y | PY | Y | Y | PY | PY | NI | PY | Y | N | PN | Y | N | N | N | NI |
| A120 | PN | PN | N | PN | Y | PY | Y | Y | PY | PY | NI | PY | Y | N | PN | Y | N | N | N | NI |
| A120 | PN | PN | N | PN | Y | PY | Y | Y | PY | PY | NI | PY | Y | N | PN | Y | N | N | N | NI |
| A120 | PN | PN | N | PN | Y | PY | Y | Y | PY | PY | NI | PY | Y | N | PN | Y | N | N | N | NI |
| A121 | N | N | N | Y | Y | N | N | N | N | N | Y | PY | N | N | N | Y | N | N | Y | Y |
| A121 | N | N | N | Y | Y | N | N | N | N | N | Y | PY | N | N | N | Y | N | N | Y | Y |
| A121 | N | N | N | N | Y | N | N | N | N | N | N | PY | N | N | N |  | N | N |  |  |
| A121 | N | N | N | Y | Y | N | N | N | N | N | Y | PY | N | N | N | Y | N | N | Y | Y |
| A121 | N | N | N | N | Y | N | N | N | N | N | N | PY | N | N | N |  | N | N |  |  |
| A133 | Y | N | PY | Y | Y | PY | Y | Y | PY | PY | N | N | N | N | N | Y | N | N | N | NI |
| A133 | Y | Y | PY | Y | Y | PY | Y | Y | PY | PY | N | N | Y | N | N |  | N | N |  |  |
| A133 | Y | N | PY | Y | Y | PY | Y | Y | PY | PY | N | N | N | N | N | Y | N | N | N | NI |
| A133 | Y | Y | PY | Y | Y | PY | Y | Y | PY | PY | N | N | Y | N | N |  | N | N |  |  |
| A133 | Y | N | PY | Y | Y | PY | Y | Y | PY | PY | N | N | N | N | N | Y | N | N | N | NI |
| A133 | Y | Y | PY | Y | Y | PY | Y | Y | PY | PY | N | N | Y | N | N |  | N | N |  |  |
| A134 | N | N | N | Y | NI | PY | Y | Y | N | PY | NI | PN | Y | N | N | N | N | N | N | NI |
| A135 | PN | N | PN | PY | Y | N | Y | Y | PY | PY | N | NI | N | N | N | Y | N | N | N | NI |
| A135 | PN | N | PN | PY | Y | N | Y | Y | PY | PY | N | NI | N | N | N | Y | N | N | N | NI |
| A135 | PN | N | PN | PY | Y | N | Y | Y | PY | PY | N | NI | N | N | N | Y | N | N | N | NI |
| A135 | PN | N | PN | PY | Y | N | Y | Y | PY | PY | N | NI | N | N | N | Y | N | N | N | NI |
| A135 | PN | N | PN | PY | Y | N | Y | Y | PY | PY | N | NI | N | N | N | Y | N | N | N | NI |
| A135 | PN | N | PN | PY | Y | N | Y | Y | PY | PY | N | NI | N | N | N | Y | N | N | N | NI |
| A136 | Y | N | N | Y | Y | Y | Y | Y | Y | PY | Y | N | N | N | N | Y | N | N | N | NI |
| A136 | Y | N | N | Y | Y | Y | Y | Y | Y | PY | Y | PY | Y | N | N |  | N | N |  |  |
| A136 | Y | N | N | Y | Y | Y | Y | Y | Y | PY | Y | PY | Y | N | N |  | N | N |  |  |
| A136 | Y | N | N | Y | Y | Y | Y | Y | Y | PY | Y | PY | Y | N | N |  | N | N |  |  |
| A137 | PN | N | PN | PY | Y | N | Y | Y | PY | PY | N | NI | N | N | N | Y | N | N | N | NI |
| A137 | PN | N | PN | PY | Y | N | Y | Y | PY | PY | N | NI | N | N | N | Y | N | N | N | NI |
| A137 | PN | N | PN | PY | Y | N | Y | Y | PY | PY | N | NI | N | N | N | Y | N | N | N | NI |
| A137 | PN | N | PN | PY | Y | N | Y | Y | PY | PY | N | NI | N | N | N | Y | N | N | N | NI |
| A137 | PN | N | PN | PY | Y | N | Y | Y | PY | PY | N | NI | N | N | N | Y | N | N | N | NI |
| A137 | PN | N | PN | PY | Y | N | Y | Y | PY | PY | N | NI | N | N | N | Y | N | N | N | NI |
| A137 | PN | N | PN | PY | Y | N | Y | Y | PY | PY | N | NI | N | N | N | Y | N | N | N | NI |
| A137 | PN | N | PN | PY | Y | N | Y | Y | PY | PY | N | NI | N | N | N | Y | N | N | N | NI |
| A137 | PN | N | PN | PY | Y | N | Y | Y | PY | PY | N | NI | N | N | N | Y | N | N | N | NI |
| A137 | PN | N | PN | PY | Y | N | Y | Y | PY | PY | N | NI | N | N | N | Y | N | N | N | NI |
| A137 | PN | N | PN | PY | Y | N | Y | Y | PY | PY | N | NI | N | N | N | Y | N | N | N | NI |
| A137 | PN | N | PN | PY | Y | N | Y | Y | PY | PY | N | NI | N | N | N | Y | N | N | N | NI |
| A137 | PN | N | PN | PY | Y | N | Y | Y | PY | PY | N | NI | N | N | N | Y | N | N | N | NI |
| A137 | PN | N | PN | PY | Y | N | Y | Y | PY | PY | N | NI | N | N | N | Y | N | N | N | NI |
| A137 | PN | N | PN | PY | Y | N | Y | Y | PY | PY | N | NI | N | N | N | Y | N | N | N | NI |
| A137 | PN | N | PN | PY | Y | N | Y | Y | PY | PY | N | NI | N | N | N | Y | N | N | N | NI |
| A137 | PN | N | PN | PY | Y | N | Y | Y | PY | PY | N | NI | N | N | N | Y | N | N | N | NI |
| A137 | PN | N | PN | PY | Y | N | Y | Y | PY | PY | N | NI | N | N | N | Y | N | N | N | NI |
| A137 | PN | N | PN | PY | Y | N | Y | Y | PY | PY | N | NI | N | N | N | Y | N | N | N | NI |
| A137 | PN | N | PN | PY | Y | N | Y | Y | PY | PY | N | NI | N | N | N | Y | N | N | N | NI |
| A137 | PN | N | PN | PY | Y | N | Y | Y | PY | PY | N | NI | N | N | N | Y | N | N | N | NI |
| A137 | PN | N | PN | PY | Y | N | Y | Y | PY | PY | N | NI | N | N | N | Y | N | N | N | NI |
| A137 | PN | N | PN | PY | Y | N | Y | Y | PY | PY | N | NI | N | N | N | Y | N | N | N | NI |
| A137 | PN | N | PN | PY | Y | N | Y | Y | PY | PY | N | NI | N | N | N | Y | N | N | N | NI |
| A137 | PN | N | PN | PY | Y | N | Y | Y | PY | PY | N | NI | N | N | N | Y | N | N | N | NI |
| A139 | Y | Y | Y | Y | Y | Y | Y | PY | Y | PY | PY | PY | Y | N | N |  | N | N |  |  |
| A139 | Y | Y | Y | Y | Y | Y | Y | PY | Y | PY | PY | PY | Y | N | N |  | N | N |  |  |
| A139 | Y | Y | Y | Y | Y | Y | Y | PY | Y | PY | PY | PY | Y | N | N |  | N | N |  |  |
| A144 | Y | PY | PY | Y | Y | PY | Y | Y | PY | PY | Y | PY | N | NI | NI | N | N | N | N | NI |
| A145 | Y | N | N | Y | Y | PY | Y | Y | PY | PY | Y | N | PN | N | N | Y | Y | N | PN | NI |
| A146 | Y | N | N | Y | Y | PY | N | N | PY | PY | Y | N | PY | N | N | N | N | N | N | NI |
| A146 | Y | N | N | Y | Y | PY | N | N | PY | PY | Y | N | PY | N | N | N | N | N | N | NI |
| A146 | Y | N | N | Y | Y | PY | N | N | PY | PY | Y | N | PY | N | N | N | N | N | N | NI |
| A146 | Y | N | Y | Y | Y | PY | N | N | PY | PY | Y | N | PY | N | N | N | N | N | N | NI |
| A146 | Y | N | N | Y | Y | PY | N | N | PY | PY | Y | N | PY | N | N | N | N | N | N | NI |

All data collected about each study and model is available via the following link (tinyurl.com/442f4bdz).

**Table S8:**

Individual question risk of bias ratings grouped as positive, negative or no information and reported for developed models by type of methodology used

|  | **Title of domain /**  **Screening Question** | **Regression methods (n=97)** | | |  | **Flexible machine learning methods (n=45)** | | |  | **Ensemble machine learning methods (n=35)** | | |
| --- | --- | --- | --- | --- | --- | --- | --- | --- | --- | --- | --- | --- |
|  | **Overall risk of bias** | **LOW** | **HIGH** | **UNCLEAR** |  | **LOW** | **HIGH** | **UNCLEAR** |  | **LOW** | **HIGH** | **UNCLEAR** |
|  |  | n = 0,  0% | n = 97,  100% | n = 0,  0% |  | n = 0,  0% | n = 45,  100% | n = 0,  0% |  | n=0,  0% | n = 35,  100% | n = 0,  0% |
| **Domain 1** | **Participants** | **LOW** | **HIGH** | **UNCLEAR** |  | **LOW** | **HIGH** | **UNCLEAR** |  | **LOW** | **HIGH** | **UNCLEAR** |
|  |  | n = 31,  32% | n = 49,  51% | n = 17,  17% |  | n = 7,  16% | n = 28,  62% | n = 10,  22% |  | n = 7,  20% | n = 20,  57% | n = 8,  23% |
| **Screening questions:** | | **Y or PY** | **N or PN** | **NI** |  | **Y or PY** | **N or PN** | **NI** |  | **Y or PY** | **N or PN** | **NI** |
| 1.1 | Were appropriate data sources used, e.g., cohort, randomized controlled trial, or nested case-control study data? | n = 58,  60% | n = 33,  34% | n = 6,  6% |  | n = 12,  27% | n = 23,  51% | n = 10,  22% |  | n = 10,  28% | n = 23,  66% | n = 2,  6% |
| 1.2 | Were all inclusions and exclusions of participants appropriate? | n = 45,  47% | n = 42,  43% | n = 10,  10% |  | n = 7,  16% | n = 25,  55% | n = 13,  29% |  | n = 7,  20% | n = 24,  69% | n = 4,  11% |
|  |  |  |  |  |  |  |  |  |  |  |  |  |
| **Domain 2** | **Predictors** | **LOW** | **HIGH** | **UNCLEAR** |  | **LOW** | **HIGH** | **UNCLEAR** |  | **LOW** | **HIGH** | **UNCLEAR** |
|  |  | n = 33,  34% | n = 49,  51% | n = 15,  15% |  | n = 8,  18% | n = 19,  42% | n = 18,  40% |  | n = 7,  20% | n = 9,  26% | n = 19,  54% |
| **Screening questions:** | | **Y or PY** | **N or PN** | **NI** |  | **Y or PY** | **N or PN** | **NI** |  | **Y or PY** | **N or PN** | **NI** |
| 2.1 | Were predictors defined and assessed in a similar way for all participants? | n = 36,  37% | n = 55,  57% | n = 6,  6% |  | n = 9,  20% | n = 26,  58% | n = 10,  22% |  | n = 7,  20% | n = 26,  74% | n = 2,  6% |
| 2.2 | Were predictor assessments made without knowledge of outcome data? | n = 83,  86% | n = 9,  9% | n = 5,  5% |  | n = 20,  44% | n = 18,  40% | n = 7,  16% |  | n = 17,  48% | n = 15,  43% | n = 3,  9% |
| 2.3 | Are all predictors available at the time the model is intended to be used? | n = 94,  97% | n = 2,  2% | n = 1,  1% |  | n = 38,  85% | n = 6,  13% | n = 1,  2% |  | n = 33,  94% | n = 2,  6% | n = 0,  0% |
|  |  |  |  |  |  |  |  |  |  |  |  |  |
| **Domain 3** | **Outcome** | **LOW** | **HIGH** | **UNCLEAR** |  | **LOW** | **HIGH** | **UNCLEAR** |  | **LOW** | **HIGH** | **UNCLEAR** |
|  |  | n = 45,  46% | n = 48,  50% | n = 4,  4% |  | n = 12,  27% | n = 32,  71% | n = 1,  2% |  | n = 11,  31% | n = 24,  69% | n = 0,  0% |
| **Screening questions:** | | **Y or PY** | **N or PN** | **NI** |  | **Y or PY** | **N or PN** | **NI** |  | **Y or PY** | **N or PN** | **NI** |
| 3.1 | Was the outcome determined appropriately? | n = 64,  66% | n = 30,  31% | n = 3,  3% |  | n = 14,  31% | n = 25,  56% | n = 6,  13% |  | n = 11,  31% | n = 22,  63% | n = 2,  6% |
| 3.2 | Was a prespecified or standard outcome definition used? | n = 88,  91% | n = 9,  9% | n = 0,  0% |  | n = 43,  96% | n = 2,  4% | n = 0,  0% |  | n = 35,  100% | n = 0,  0% | n = 0,  0% |
| 3.3 | Were predictors excluded from the outcome definition? | n = 86,  89% | n = 11,  11% | n = 0,  0% |  | n = 44,  98% | n = 1,  2% | n = 0,  0% |  | n = 35,  100% | n = 0,  0% | n = 0,  0% |
|  | **Title of domain /**  **Screening Question** | **Regression methods (n=97)** | | |  | **Flexible machine learning methods (n=45)** | | |  | **Ensemble machine learning methods (n=35)** | | |
| **Screening questions:** | | **Y or PY** | **N or PN** | **NI** |  | **Y or PY** | **N or PN** | **NI** |  | **Y or PY** | **N or PN** | **NI** |
| 3.4 | Was the outcome defined and determined in a similar way for all participants? | n = 65,  67% | n = 25,  26% | n = 7,  7% |  | n = 19,  42% | n = 16,  36% | n = 10,  22% |  | n = 24,  68% | n = 9,  26% | n = 2,  6% |
| 3.5 | Was the outcome determined without knowledge of predictor information? | n = 40,  41% | n = 20,  21% | n = 37,  38% |  | n = 17,  38% | n = 15,  33% | n = 13,  29% |  | n = 19,  54% | n = 13,  37% | n = 3,  9% |
| 3.6 | Was the time interval between predictor assessment and outcome determination appropriate? | n = 59,  61% | n = 34,  35% | n = 4,  4% |  | n = 18,  40% | n = 20,  44% | n = 7,  16% |  | n = 11,  31% | n = 17,  49% | n = 7,  20% |
|  |  |  |  |  |  |  |  |  |  |  |  |  |
| **Domain 4** | **Analysis** | **LOW** | **HIGH** | **UNCLEAR** |  | **LOW** | **HIGH** | **UNCLEAR** |  | **LOW** | **HIGH** | **UNCLEAR** |
|  |  | n = 0,  0% | n = 97,  100% | n = 0,  0% |  | n = 0,  0% | n = 45,  100% | n = 0,  0% |  | n = 0,  0% | n = 35,  100% | n = 0,  0% |
| **Screening questions:** | | **Y or PY** | **N or PN** | **NI** |  | **Y or PY** | **N or PN** | **NI** |  | **Y or PY** | **N or PN** | **NI** |
| 4.1 | Were there a reasonable number of participants with the outcome? | n = 50,  52% | n = 39,  40% | n = 8,  8% |  | n = 18,  40% | n = 17,  38% | n = 10,  22% |  | n = 16,  46% | n = 6,  17% | n = 13,  37% |
| 4.2 | Were continuous and categorical predictors handled appropriately? | n = 21,  22% | n = 72,  74% | n = 4,  4% |  | n = 7,  16% | n = 27,  60% | n = 11,  24% |  | n = 10,  28% | n = 23,  66% | n = 2,  6% |
| 4.3 | Were all enrolled participants included in the analysis? | n = 0,  0% | n = 82,  85% | n = 15,  15% |  | n = 0,  0% | n = 31,  69% | n = 14,  31% |  | n = 0,  0% | n = 31,  89% | n = 4,  11% |
| 4.4 | Were participants with missing data handled appropriately? | n = 2,  2% | n = 75,  77% | n = 20,  21% |  | n = 7,  15% | n = 26,  58% | n = 12,  27% |  | n = 4,  11% | n = 29,  83% | n = 2,  6% |
| 4.5 | Was selection of predictors based on univariable analysis avoided? | n = 36,  37% | n = 44,  45% | n = 17,  18% |  | n = 24,  53% | n = 14,  31% | n = 7,  16% |  | n = 28,  80% | n = 4,  11% | n = 3,  9% |
| 4.7 | Were relevant model performance measures evaluated appropriately? | n = 21,  22% | n = 76,  78% | n = 0,  0% |  | n = 0,  0% | n = 45,  100% | n = 0,  0% |  | n = 0,  0% | n = 35,  100% | n = 0,  0% |
| 4.8 | Were model overfitting and optimism in model performance accounted for? | n = 13,  13% | n = 63,  65% | n = 21,  22% |  | n = 8,  18% | n = 26,  58% | n = 11,  24% |  | n = 6,  17% | n = 22,  63% | n = 7,  20% |
| 4.9 | Do predictors and their assigned weights in the final model correspond to the results from the reported multivariable analysis? | n = 31,  32% | n = 10,  10% | n = 56,  58% |  | n = 0,  0% | n = 2,  4% | n = 43,  96% |  | n = 0,  0% | n = 5,  14% | n = 30,  86% |
|  |  |  |  |  |  |  |  |  |  |  |  |  |
| **Sensitivity analysis:** | | **LOW** | **HIGH** | **UNCLEAR** |  | **LOW** | **HIGH** | **UNCLEAR** |  | **LOW** | **HIGH** | **UNCLEAR** |
| ***Risk of bias rating not including analysis domain:*** | | *n = 13,*  *13%* | *n = 80,*  *83%* | *n = 4,*  *4%* |  | *n = 2,*  *4%* | *n = 43,*  *96%* | *n = 0,*  *0%* |  | *n = 4,*  *11%* | *n = 31,*  *89%* | *n = 0,*  *0%* |

Please note: 3 models assessed did not report method, and are thus not included in this table

**Table S9:**

Individual question ratings for the top ranking developed and validated model from all articles, conducted as a sensitivity analysis to assess whether articles with a large number of developed models had a disproportionate effect on the results.

|  |  | **Developed models (n=50)**  **n, %** | | | | |  | **Validated models (n=27)**  **n, %** | | | | |
| --- | --- | --- | --- | --- | --- | --- | --- | --- | --- | --- | --- | --- |
|  | **Question** | **Y** | **PY** | **PN** | **N** | **NI** |  | **Y** | **PY** | **PN** | **N** | **NI** |
| 1.1 | Were appropriate data sources used, e.g., cohort, randomized controlled trial, or nested case control study data? | n = 34,  68% | n = 1,  2% | n = 7,  14% | n = 6,  12% | n = 2,  4% |  | n = 20,  74% | n = 2,  7% | n = 1,  4% | n = 4,  15% | n = 0,  0% |
| 1.2 | Were all inclusions and exclusions of participants appropriate? | n = 13,  26% | n = 7,  14% | n = 3,  6% | n = 20,  40% | n = 7,  14% |  | n = 7,  26% | n = 5,  19% | n = 0,  0% | n = 12,  44% | n = 3,  11% |
|  |  |  |  |  |  |  |  |  |  |  |  |  |
| 2.1 | Were predictors defined and assessed in a similar way for all participants? | n = 14,  28% | n = 8,  16% | n = 9,  18% | n = 17,  34% | n = 2,  4% |  | n = 14,  52% | n = 6,  22% | n = 1,  4% | n = 5,  18% | n = 1,  4% |
| 2.2 | Were predictor assessments made without knowledge of outcome data? | n = 33,  66% | n = 3,  6% | n = 5,  10% | n = 5,  10% | n = 4,  8% |  | n = 24,  89% | n = 0,  0% | n = 0,  0% | n = 1,  4% | n = 2,  7% |
| 2.3 | Are all predictors available at the time the model is intended to be used? | n = 47,  94% | n = 0,  0% | n = 0,  0% | n = 1,  2% | n = 2,  4% |  | n = 27,  100% | n = 0,  0% | n = 0,  0% | n = 0,  0% | n = 0,  0% |
|  |  |  |  |  |  |  |  |  |  |  |  |  |
| 3.1 | Was the outcome determined appropriately? | n = 14,  28% | n = 20,  40% | n = 4,  8% | n = 10,  20% | n = 2,  4% |  | n = 8,  30% | n = 15,  55% | n = 1,  4% | n = 2,  7% | n = 1,  4% |
| 3.2 | Was a prespecified or standard outcome definition used? | n = 43,  86% | n = 2,  4% | n = 0,  0% | n = 5,  10% | n = 0,  0% |  | n = 23,  85% | n = 1,  4% | n = 0,  0% | n = 2,  7% | n = 1,  4% |
| 3.3 | Were predictors excluded from the outcome definition? | n = 46,  92% | n = 0,  0% | n = 0,  0% | n = 4,  8% | n = 0,  0% |  | n = 24,  89% | n = 1,  4% | n = 0,  0% | n = 2,  7% | n = 0,  0% |
| 3.4 | Was the outcome defined and determined in a similar way for all participants? | n = 7,  14% | n = 26,  52% | n = 6,  12% | n = 8,  16% | n = 3,  6% |  | n = 6,  22% | n = 17,  63% | n = 1,  4% | n = 3,  11% | n = 0,  0% |
| 3.5 | Was the outcome determined without knowledge of predictor information? | n = 0,  0% | n = 19,  38% | n = 7,  14% | n = 8,  16% | n = 16,  32% |  | n = 0,  0% | n = 16,  59% | n = 1,  4% | n = 1,  4% | n = 9,  33% |
| 3.6 | Was the time interval between predictor assessment and outcome determination appropriate? | n = 33,  66% | n = 1,  2% | n = 3,  6% | n = 7,  14% | n = 6,  12% |  | n = 19,  70% | n = 2,  7% | n = 1,  4% | n = 4,  15% | n = 1,  4% |
|  |  |  |  |  |  |  |  |  |  |  |  |  |
|  | **Question** | **Developed models (n=50)**  **n, %** | | | | |  | **Validated models (n=27)**  **n, %** | | | | |
|  |  | **Y** | **PY** | **PN** | **N** | **NI** |  | **Y** | **PY** | **PN** | **N** | **NI** |
| 4.1 | Were there a reasonable number of participants with the outcome? | n = 15,  30% | n = 13,  26% | n = 6,  12% | n = 14,  28% | n = 2,  4% |  | n = 9,  33% | n = 10,  37% | n = 1,  4% | n = 7,  26% | n = 0,  0% |
| 4.2 | Were continuous and categorical predictors handled appropriately? | n = 10,  20% | n = 3,  6% | n = 1,  2% | n = 30,  60% | n = 6,  12% |  | n = 23,  85% | n = 0,  0% | n = 0,  0% | n = 3,  11% | n = 1,  4% |
| 4.3 | Were all enrolled participants included in the analysis? | n = 0,  0% | n = 0,  0% | n = 1,  2% | n = 38,  76% | n = 11,  22% |  | n = 1,  4% | n = 0,  0% | n = 0,  0% | n = 18,  66% | n = 8,  30% |
| 4.4 | Were participants with missing data handled appropriately? | n = 1,  2% | n = 2,  4% | n = 2,  4% | n = 32,  64% | n = 13,  26% |  | n = 0,  0% | n = 1,  4% | n = 0,  0% | n = 15,  55% | n = 11,  41% |
| 4.5 | Was selection of predictors based on univariable analysis avoided? | n = 20,  40% | n = 2,  4% | n = 5,  10% | n = 16,  32% | n = 7,  14% |  |  |  |  |  |  |
| 4.6 | Were complexities in the data (e.g. censoring, competing risks, sampling of control participants) accounted for appropriately? | n = 11,  22% | n = 0,  0% | n = 2,  4% | n = 30,  60% | n = 7,  14% |  | n = 2,  7% | n = 0,  0% | n = 1,  4% | n = 19,  70% | n = 5,  19% |
| 4.7 | Were relevant model performance measures evaluated appropriately? | n = 5,  10% | n = 2,  4% | n = 2,  4% | n = 41,  82% | n = 0,  0% |  | n = 3,  11% | n = 0,  0% | n = 1,  4% | n = 23,  85% | n = 0,  0% |
| 4.8 | Were model overfitting and optimism in model performance accounted for? | n = 6,  12% | n = 1,  2% | n = 4,  8% | n = 26,  52% | n = 13,  26% |  |  |  |  |  |  |
| 4.9 | Do predictors and their assigned weights in the final model correspond to the results from the reported multivariable analysis? | n = 12,  24% | n = 2,  4% | n = 1,  2% | n = 5,  10% | n = 30,  60% |  |  |  |  |  |  |

**Table S10:**

Individual question with grouped ratings for the top ranking developed and validated model from all articles, conducted as a sensitivity analysis to assess whether articles with a large number of developed models had a disproportionate effect on the results.

|  | **Title of domain /**  **screening question** | **Developed models (n=50)**  **n, %** | | |  | **Validated models (n=27)**  **n, %** | | |
| --- | --- | --- | --- | --- | --- | --- | --- | --- |
| **Overall risk of bias** | | **LOW** | **HIGH** | **UNCLEAR** |  | **LOW** | **HIGH** | **UNCLEAR** |
|  |  | n = 0,  0% | n = 50,  100% | n = 0,  0% |  | n = 0,  0% | n = 27,  100% | n = 0,  0% |
|  |  |  |  |  |  |  |  |  |
| **Domain**  **1** | **Participants** | **LOW** | **HIGH** | **UNCLEAR** |  | **LOW** | **HIGH** | **UNCLEAR** |
|  |  | n = 17,  34% | n = 28,  56% | n = 5,  10% |  | n = 10,  37% | n = 15,  56% | n = 2,  7% |
| **Screening questions:** | | **Yes /**  **probably yes** | **No /**  **probably no** | **No**  **information** |  | **Yes /**  **probably yes** | **No /**  **probably no** | **No**  **information** |
| **1.1** | Were appropriate data sources used, e.g., cohort, randomized controlled trial, or nested case-control study data? | n = 35,  70% | n = 13,  26% | n = 2,  4% |  | n = 22,  81% | n = 5,  19% | n = 0,  0% |
| **1.2** | Were all inclusions and exclusions of participants appropriate? | n = 20,  40% | n = 23,  46% | n = 7,  14% |  | n = 12,  44% | n = 12,  44% | n = 3,  12% |
|  | 51 |  |  |  |  |  |  |  |
| **Domain**  **2** | **Predictors** | **LOW** | **HIGH** | **UNCLEAR** |  | **LOW** | **HIGH** | **UNCLEAR** |
|  |  | n = 20,  40% | n = 22,  44% | n = 8,  16% |  | n = 21,  78% | n = 5,  18% | n = 1,  4% |
| **Screening questions:** | | **Yes /**  **probably yes** | **No /**  **probably no** | **No**  **information** |  | **Yes /**  **probably yes** | **No /**  **probably no** | **No**  **information** |
| **2.1** | Were predictors defined and assessed in a similar way for all participants? | n = 22,  44% | n = 26,  52% | n = 2,  4% |  | n = 20,  74% | n = 6,  22% | n = 1,  4% |
| **2.2** | Were predictor assessments made without knowledge of outcome data? | n = 36,  72% | n = 10,  20% | n = 4,  8% |  | n = 24,  89% | n = 1,  4% | n = 2,  7% |
| **2.3** | Are all predictors available at the time the model is intended to be used? | n = 47,  94% | n = 1,  2% | n = 2,  4% |  | n = 27,  100% | n = 0,  0% | n = 0,  0% |
|  |  |  |  |  |  |  |  |  |
| **Domain**  **3** | **Outcome** | **LOW** | **HIGH** | **UNCLEAR** |  | **LOW** | **HIGH** | **UNCLEAR** |
|  |  | n = 27,  54% | n = 20,  40% | n = 3,  6% |  | n = 19,  70% | n = 5,  19% | n = 3,  11% |
| **Screening questions:** | | **Yes /**  **probably yes** | **No /**  **probably no** | **No**  **information** |  | **Yes /**  **probably yes** | **No /**  **probably no** | **No**  **information** |
| **3.1** | Was the outcome determined appropriately? | n = 34,  68% | n = 14,  28% | n = 2,  4% |  | n = 23,  85% | n = 3,  11% | n = 1,  4% |
| **3.2** | Was a prespecified or standard outcome definition used? | n = 45,  90% | n = 5,  10% | n = 0,  0% |  | n = 24,  89% | n = 2,  7% | n = 1,  4% |
| **3.3** | Were predictors excluded from the outcome definition? | n = 46,  92% | n = 4,  8% | n = 0,  0% |  | n = 25,  93% | n = 2,  7% | n = 0,  0% |
|  | **Title of domain /**  **screening question** | **Developed models (n=50)**  **n, %** | | |  | **Validated models (n=27)**  **n, %** | | |
| **Domain**  **3** | **Outcome** | **Yes /**  **probably yes** | **No /**  **probably no** | **No**  **information** |  | **Yes /**  **probably yes** | **No /**  **probably no** | **No**  **information** |
| **3.4** | Was the outcome defined and determined in a similar way for all participants? | n = 33,  66% | n = 14,  28% | n = 3,  6% |  | n = 23,  85% | n = 4,  15% | n = 0,  0% |
| **3.5** | Was the outcome determined without knowledge of predictor information? | n = 19,  38% | n = 15,  30% | n = 16,  32% |  | n = 16,  59% | n = 2,  8% | n = 9,  33% |
| **3.6** | Was the time interval between predictor assessment and outcome determination appropriate? | n = 34,  68% | n = 10,  20% | n = 6,  12% |  | n = 21,  78% | n = 5,  18% | n = 1,  4% |
|  |  |  |  |  |  |  |  |  |
| **Domain**  **4** | **Analysis** | **LOW** | **HIGH** | **UNCLEAR** |  | **LOW** | **HIGH** | **UNCLEAR** |
|  |  | n = 0,  0% | n = 50,  100% | n = 0,  0% |  | n = 0,  0% | n = 27,  100% | n = 0,  0% |
| **Screening questions:** | | **Yes /**  **probably yes** | **No /**  **probably no** | **No**  **information** |  | **Yes /**  **probably yes** | **No /**  **probably no** | **No**  **information** |
| **4.1** | Were there a reasonable number of participants with the outcome? | n = 28,  56% | n = 20,  40% | n = 2,  4% |  | n = 19,  70% | n = 8,  30% | n = 0,  0% |
| **4.2** | Were continuous and categorical predictors handled appropriately? | n = 13,  26% | n = 31,  62% | n = 6,  12% |  | n = 23,  85% | n = 3,  11% | n = 1,  4% |
| **4.3** | Were all enrolled participants included in the analysis? | n = 0,  0% | n = 39,  78% | n = 11,  22% |  | n = 1,  4% | n = 18,  67% | n = 8,  29% |
| **4.4** | Were participants with missing data handled appropriately? | n = 3,  6% | n = 34,  68% | n = 13,  26% |  | n = 1,  4% | n = 15,  55% | n = 11,  41% |
| **4.5** | Was selection of predictors based on univariable analysis avoided? | n = 22,  44% | n = 21,  42% | n = 7,  14% |  |  |  |  |
| **4.6** | Were complexities in the data (e.g. censoring, competing risks, sampling of control participants) accounted for appropriately? | n = 11,  22% | n = 32,  64% | n = 7,  14% |  | n = 2,  7% | n = 20,  74% | n = 5,  19% |
| **4.7** | Were relevant model performance measures evaluated appropriately? | n = 7,  14% | n = 43,  86% | n = 0,  0% |  | n = 3,  11% | n = 24,  89% | n = 0,  0% |
| **4.8** | Were model overfitting and optimism in model performance accounted for? | n = 7,  14% | n = 30,  60% | n = 13,  26% |  |  |  |  |
| **4.9** | Do predictors and their assigned weights in the final model correspond to the results from the reported multivariable analysis? | n = 14,  28% | n = 6,  12% | n = 30,  60% |  |  |  |  |
|  |  |  |  |  |  |  |  |  |
| **Sensitivity analysis:** | | **LOW** | **HIGH** | **UNCLEAR** |  | **LOW** | **HIGH** | **UNCLEAR** |
| ***Risk of bias rating not including analysis domain:*** | | n = 8,  16% | n = 40,  80% | n = 2,  4% |  | n = 8,  29% | n = 1,  4% | n = 18,  67% |

**Supplementary Checklists**

Checklist 1: TRIPOD-SRMA checklist for reporting systematic reviews of prediction model studies

| Section and topic | Item No | Checklist item | Page of Manuscript |
| --- | --- | --- | --- |
| **Title** | | | |
| Title | 1 | Identify the report as a systematic review or meta-analysis (or both) of diagnostic or prognostic model studies. Specify the target population and outcome(s) predicted as relevant to the review question. | Page 1 |
| **Abstract** | | | |
| Abstract | 2 | See the TRIPOD-SRMA checklist for abstracts | Page 2  (checklist below) |
| **Introduction** | | | |
| Rationale | 3 | Describe the rationale for the review in the context of existing knowledge. | Page 4 |
| Objectives | 4 | Provide an explicit statement of the objective(s) being addressed with reference to: target population, index and comparator models (as relevant), outcome(s), time (prediction horizon and intended moment of using the model), and setting. | Page 4 |
| **Methods** | | | |
| Study eligibility criteria | 5 | Specify study characteristics used as eligibility criteria, including any prediction models of specific interest, and whether development or validation studies (or both) were eligible. | Page 5 |
| Information sources | 6 | Specify all databases, registers, websites, organisations, reference lists, and other sources searched or consulted to identify studies. Specify the date when each source was last searched or consulted. | Page 5 |
| Search strategy | 7 | Present the full search strategies for all databases, registers, and websites, including any filters and limits used. | Page 5 / Table S1 |
| Study selection process | 8 | Specify the methods used to decide whether a study met the inclusion criteria of the review, including how many reviewers screened each record and each report retrieved, whether they worked independently, and if applicable, details of automation tools used in the process. | Page 5 |
| Data collection process | 9 | Specify the methods used to collect data from study reports, including how many reviewers collected data from each report, whether they worked independently, any processes for obtaining or confirming data from study investigators, and if applicable, details of automation tools used in the process. | Page 6 |
| Data Items | 10a | List and define all items for which data were sought from each study. | Page 6 / Table S2 /  Table S3 |
|  | 10b | State the model performance measures that were sought (eg, measures of calibration, discrimination, overall model fit, clinical utility). | Page 5 |
|  | 10c | Describe how any desired but unreported data items (items 10a, 10b) were handled (eg, contacted authors, calculated from other reported information). | Page 5 |
| Risk of bias and applicability assessment | 11 | Specify the methods used to assess risk of bias in the included studies and their applicability to the review question. This should be done separately for each model development and validation. Include details of any tool(s) used, how many reviewers assessed each study and whether they worked independently. | Page 4 to 5 |
| Synthesis methods | 12a | Describe any methods for synthesising estimates of performance measures for each model. If meta-analysis was carried out, describe the methods used, including any transformations of data before pooling, how any heterogeneity in model performance was quantified and handled, and software package(s) used. | Page 5 |
|  | 12b | Describe any methods used to explore possible causes of heterogeneity in model performance (eg, subgroup analysis, meta-regression), including whether or not they were planned. | Page 4 |
|  | 12c | Describe any sensitivity analyses conducted to assess robustness of the synthesised results. | Page 8 |
| Certainty assessment | 13 | Describe any methods used to assess certainty (or confidence) in the body of evidence for a prediction model. | Page 4 |
| **Results** | | | |
| Study selection | 14 | Describe the results of the search and selection process, from the number of records identified in the search to the number of studies and models included in the review, ideally using a flow diagram. | Page 6 / Figure 1 /  Table S4 |
| Study and model characteristics | 15 | Present study characteristics and model details extracted (as per item 10a), and cite the study reports. | Page 6 /  Figure 1 |
| Risk of bias and applicability | 16 | Present results of risk of bias and applicability assessment. This should be done separately for each model development and validation in each included study. | Table 2 / Figure 2 / Table S6 / Table S7 / Figure S4 / Table S8 / Table S9 / Table S10 |
| Results of model performance in individual studies | 17 | Present performance estimates and confidence intervals for each model and all evaluations, including whether they relate to the internal or external validation performance. If internal, give details of the method. | Figure S4 |
| Results of syntheses | 18a | Present the results of any synthesis of model performance, together with details of which study estimates contributed. If meta-analysis was carried out, then for each model and performance measure, present summary results, confidence/credible intervals, and measures of heterogeneity. Forest plots may be useful. | Table S7 |
|  | 18b | For each model, present results of all investigations of possible causes of heterogeneity in model performance. | Table S7 |
|  | 18c | Present results of all sensitivity analyses conducted to assess the robustness of the synthesised results. | Page 8 / Table 2 |
| Certainty of evidence | 19 | Present any assessments of certainty (or confidence) in the body of evidence for each prediction model of interest. | Page 7 |
| **Discussion** | | | |
| Summary of evidence | 20 | Summarise the main findings including the strengths and limitations of the evidence. | Page 9 / Page 10 |
| Limitations | 21 | Discuss the strengths and limitations of the review process. | Page 10 |
| Implications | 22 | Discuss implications of the results in the context of other evidence and for practice, policy, and future research. | Page 9 / Page 10 |
| **Other information** | | | |
| Registration and protocol | 23a | Provide registration information for the review, including register name and registration number, or state that the review was not registered. | Page 5 |
|  | 23b | Indicate where the review protocol can be accessed, or state that a protocol was not prepared. | Page 5 |
|  | 23c | Describe and explain any amendments to information provided at registration or in the protocol. | N/A  Reference 24 – protocol not changed |
| Support | 24 | Describe sources of financial or non-financial support for the review, and the role of the funders or sponsors in the review. | Page 1 |
| Competing interests | 25 | Declare any competing interests of review authors. | Page 1 |
| Availability of data, code, and other materials | 26 | Report which of the following are publicly available and where they can be found: template data collection forms; data extracted from included studies; data used for all analyses; analytic code; any other materials used in the review. | Page 14 |

Checklist 2: TRIPOD-SRMA checklist for abstracts

| Section and topic | Item No | Checklist item | Present in abstract (page 2) |
| --- | --- | --- | --- |
| **Abstract Title** | | | |
| Title | 1 | Identify the report as a systematic review or meta-analysis (or both) of diagnostic or prognostic model studies. Specify the target population and outcome(s) predicted as relevant to the review question. | ✓ |
| **Abstract Background** | | | |
| Objectives | 2 | Provide an explicit statement of the main objective(s) being addressed with reference to: target population, index and comparator models (as relevant), outcome(s), time (prediction horizon and intended moment of using the model), and setting. | ✓ |
| **Abstract Methods** | | | |
| Study eligibility criteria | 3 | Specify study characteristics used as eligibility criteria, including any prediction models of specific interest, and whether development or validation studies (or both) were eligible. | ✓ |
| Information sources | 4 | Specify the information sources (eg, databases, registers) used to identify studies and the date when each was last searched. | ✓ |
| Risk of bias and applicability | 5 | Specify the methods used to assess risk of bias and applicability in the included studies. | ✓ |
| Synthesis methods | 6 | Specify the methods used to synthesise performance measures for each model of interest. | ✓ |
| **Abstract Results** | | | |
| Included studies | 7 | Give the total number of included studies and models, and summarise relevant study characteristics and model details. | ✓ |
| Results of syntheses | 8 | Present results for each of the main models of interest. If meta-analysis was used to synthesise study estimates of model performance, report the summary result and confidence/credible interval for each performance measure, together with the number of study estimates contributing. | ✓ |
| **Abstract Discussion** | | | |
| Limitations of evidence | 9 | Provide a brief summary of the limitations of the evidence included in the review. | ✓ |
| Interpretation | 10 | Provide a general interpretation of the results and important implications for research and practice. | ✓ |

Supplementary Material References

1. Wolff RF, Moons KGM, Riley RD, Whiting PF, Westwood M, Collins GS, et al. PROBAST: A Tool to Assess the Risk of Bias and Applicability of Prediction Model Studies. Ann Intern Med. 2019;170(1):51-8.

2. Moons KGM, Wolff RF, Riley RD, Whiting PF, Westwood M, Collins GS, et al. PROBAST: A Tool to Assess Risk of Bias and Applicability of Prediction Model Studies: Explanation and Elaboration. Ann Intern Med. 2019;170(1):W1-W33.

3. Feng YD, Wang J, Tao ZB, Jiang HK. Development and validation of a nomogram to predict poor short-term response to recombinant human growth hormone treatment in children with growth disorders. J Endocrinol Invest. 2023;46(7):1343-59.

4. Liu Z, Han N, Su T, Ji Y, Bao H, Zhou S, et al. Interpretable machine learning to identify important predictors of birth weight: A prospective cohort study. Front Pediatr. 2022;10:899954.

5. van Dommelen P, Arnaud L, Koledova E. Curve matching to predict growth in patients receiving growth hormone therapy: An interpretable & explainable method. Front Endocrinol (Lausanne). 2022;13:999077.

6. Bekele WT. Machine learning algorithms for predicting low birth weight in Ethiopia. BMC Med Inform Decis Mak. 2022;22(1):232.

7. Chen L, Fan X, Mao K, Tolba A, Alqahtani F, Ahmed AM. Study of Multidimensional and High-Precision Height Model of Youth Based on Multilayer Perceptron. Comput Intell Neurosci. 2022;2022:7843455.

8. Iwama N, Obara T, Ishikuro M, Murakami K, Ueno F, Noda A, et al. Risk scores for predicting small for gestational age infants in Japan: The TMM birthree cohort study. Sci Rep. 2022;12(1):8921.

9. Feng Y, Zheng H, Fang D, Mei S, Zhong W, Zhang G. Prediction of late-onset fetal growth restriction using a combined first- and second-trimester screening model. J Gynecol Obstet Hum Reprod. 2022;51(2):102273.

10. Ikwuezunma I, Wang K, Raymond S, Badin D, Kreulen RT, Jain A, et al. Height Gain After Spinal Fusion for Idiopathic Scoliosis: Which Model Fits Best? J Pediatr Orthop. 2022;42(9):457-61.

11. Kim HS, Oh SY, Cho GJ, Choi SJ, Hong SC, Kwon JY, et al. A Predictive Model for Large-for-Gestational-Age Infants among Korean Women with Gestational Diabetes Mellitus Using Maternal Characteristics and Fetal Biometric Parameters. J Clin Med. 2022;11(17).

12. Jing G, Huwei S, Chao C, Lei C, Ping W, Zhongzhou X, et al. A predictive model of macrosomic birth based upon real-world clinical data from pregnant women. BMC Pregnancy Childbirth. 2022;22(1):651.

13. Deval R, Saxena P, Pradhan D, Mishra AK, Jain AK. A Machine Learning-Based Intrauterine Growth Restriction (IUGR) Prediction Model for Newborns. Indian J Pediatr. 2022;89(11):1140-3.

14. Lee HS, Kum CD, Rho JG, Hwang JS. Long-term effectiveness of growth hormone therapy in children born small for gestational age: An analysis of LG growth study data. PLoS One. 2022;17(4):e0266329.

15. Tang X, Zhao Y, Liu Q, Hu D, Li G, Sun J, et al. The Effect of Risk Accumulation on Childhood Stunting: A Matched Case-Control Study in China. Front Pediatr. 2022;10:816870.

16. Wu C, Sun J, Dong X, Cai L, Deng X, Zhang F, et al. Establishment of a New Equation for Ultrasonographic Estimated Foetal Weight in Chongqing: A Prospective Study. IMR Press. 2022;49(12):271.

17. Kim YR, Park G, Joo EH, Jang JH, Ahn EH, Jung SH, et al. First-trimester screening model for small-for-gestational-age using maternal clinical characteristics, serum screening markers, and placental volume: prospective cohort study. J Matern Fetal Neonatal Med. 2022;35(25):5149-54.

18. Blue NR, Allshouse AA, Heerboth S, Grobman W, Mercer B, Shanks A, et al. Derivation and assessment of a sex-specific fetal growth standard. J Matern Fetal Neonatal Med. 2022;35(25):9913-21.

19. Wu W, Zhang B, Li D, Yan M, Deng Q, Kang Y, et al. Development and validation of nomogram for prediction of low birth weight: A large-scale cross-sectional study in northwest China. The Journal of Maternal-Fetal & Neonatal Medicine. 2022;35(25):7562-70.

20. O’Dwyer V, Russell N, McDonnell B, Sharkey L, Mulcahy C, Higgins M. Antenatal prediction of fetal macrosomia in pregnancies affected by maternal pre-gestational diabetes. The Journal of Maternal-Fetal & Neonatal Medicine. 2022;35(25):7412-6.

21. Martín-Palumbo G, Duque Alcorta M, Atanasova VB, Rego Tejeda MT, Antolin Alvarado E, Bartha JL. Prenatal prediction of very late onset small-for-gestational age newborns in low-risk pregnancies. The Journal of Maternal-Fetal & Neonatal Medicine. 2022;35(25):9816-20.

22. Bergman M, Reichman O, Farkash R, Bin-Nun A, Samueloff A, Sapir AZ, et al. Sonographic growth curves versus neonatal birthweight growth curves for the identification of fetal growth restriction. The Journal of Maternal-Fetal & Neonatal Medicine. 2022;35(23):4558-65.

23. Anum I, Zahra SS, Kumari S, Wahid A, Tahir UA, Qureshi AI. Frequency of Correct Fetal Weight Estimation by Clinical and Ultrasound Methods in Pregnant Women. Pakistan Journal of Medical & Health Sciences. 2022;16(11):251-.

24. Islam Pollob SA, Abedin MM, Islam MT, Islam MM, Maniruzzaman M. Predicting risks of low birth weight in Bangladesh with machine learning. PloS one. 2022;17(5):e0267190.

25. Zhang J, Wu X, Song Q. Analytical Comparison of Risk Prediction Models for the Onset of Macrosomia Based on Three Statistical Methods. Disease Markers. 2022;2022.

26. Xu D, Shen X, Guan H, Zhu Y, Yan M, Wu X. Prediction of small-for-gestational-age neonates at 33–39 weeks’ gestation in China: logistic regression modeling of the contributions of second-and third-trimester ultrasound data and maternal factors. BMC Pregnancy and Childbirth. 2022;22(1):661.

27. Chaliki AC, Nekkanti R, Chaliki SC, Chandra TJ. Johnsons Technique versus Hadlock – A Comparative Study to  Estimate Foetus Weight. International Journal of Pharmaceutical and Clinical Research. 2022;14(10):499-503.

28. Duncan JR, Schenone C, Dorset KM, Goedecke PJ, Tobiasz AM, Meyer NL, et al. Estimated fetal weight accuracy in pregnancies with preterm prelabor rupture of membranes by the Hadlock method. The Journal of Maternal-Fetal & Neonatal Medicine. 2022;35(9):1754-8.

29. Dieste-Pérez P, Savirón-Cornudella R, Tajada-Duaso M, Pérez-López FR, Castán-Mateo S, Sanz G, et al. Personalized Model to predict small for gestational age at delivery using fetal biometrics, maternal characteristics, and pregnancy biomarkers: a retrospective cohort study of births assisted at a Spanish hospital. Journal of Personalized Medicine. 2022;12(5):762.

30. Yuan X, Han X, Jia C, Long W, Wang H, Yu B, et al. Investigation and application of risk factors of macrosomia based on 10,396 Chinese pregnant women. Frontiers in Endocrinology. 2022;13:837816.

31. Du J, Zhang X, Chai S, Zhao X, Sun J, Yuan N, et al. Nomogram-based risk prediction of macrosomia: a case-control study. BMC Pregnancy and Childbirth. 2022;22(1):1-8.

32. Blum WF, Ranke MB, Keller E, Keller A, Barth S, de Bruin C, et al. A Novel Method for Adult Height Prediction in Children With Idiopathic Short Stature Derived From a German-Dutch Cohort. Journal of the Endocrine Society. 2022;6(7):bvac074.

33. Mejorado-Molano FJ, Sanz-Calvo ML, Posada-Ayala A, Caballo-Roig N, Gavela-Pérez T, Mahillo-Fernández I, et al. Adult Height in Girls With Idiopathic Premature Adrenarche: A Cohort Study and Design of a Predictive Model. Frontiers in Endocrinology. 2022;13:852422.

34. Mlodawski J, Wolder D, Niziurski P, Adamczyk-Gruszka O, Głuszek S, Rokita W. Birth weight prediction by Lee formula based on fractional thigh volume in term pregnancies–is it helpful? Archives of Medical Science: AMS. 2022;18(1):79.

35. Lian C, Wang Y, Bao X, Yang L, Liu G, Hao D, et al. Dynamic prediction model of fetal growth restriction based on support vector machine and logistic regression algorithm. Frontiers in Surgery. 2022;9:951908.

36. Chilyabanyama ON, Chilengi R, Simuyandi M, Chisenga CC, Chirwa M, Hamusonde K, et al. Performance of machine learning classifiers in classifying stunting among under-five children in Zambia. Children. 2022;9(7):1082.

37. Khazri HB, Shimmi SC, Parash MTH. A multivariate analysis to propose linear models for the stature estimation in the Sabahan young adult population. Plos one. 2022;17(8):e0273840.

38. McKenna M, McKenna D, Zhou M, Sonek J, Wiegand S. Prediction of Neonatal Growth Restriction in Fetuses With Gastroschisis by Early Third Trimester Ultrasonography Utilizing Contemporary Birth Weight Percentiles. Journal of Ultrasound in Medicine. 2022;42(5):997-1005.

39. Lolli L, Johnson A, Monaco M, Di Salvo V, Atkinson G, Gregson W. The percentage of mature height as a morphometric index of somatic growth: a formal scrutiny of conventional simple ratio scaling assumptions. Pediatric Exercise Science. 2022;35(2):107-15.

40. Rauh M, Rasim K, Schmidt B, Schnabel A, Köninger A. Accuracy of the sonographic determination of estimated fetal weight in anhydramnios. Archives of Gynecology and Obstetrics. 2022:1-8.

41. Huang S, Chen Z, Chen R, Zhang Z, Sun J, Chen H. Analysis of risk factors and construction of a prediction model for short stature in children. Front Pediatr. 2022;10:1006011.

42. Cheng Y, Xu H, Rissel C, Phongsavan P, Buchanan L, Taki S, et al. Estimation and feasibility of correction modelling for mother-reported child height and weight at 2 years using data from the Australian CHAT trial. Scientific Reports. 2022;12(1):21353.

43. Kiefer M, Finneran M, Ware C, Foy P, Thung S, Gabbe S, et al. Prediction of large‐for‐gestational‐age infant by fetal growth charts and hemoglobin A1c level in pregnancy complicated by pregestational diabetes. Ultrasound in Obstetrics & Gynecology. 2022;60(6):751-8.

44. Polfuss M, Liu T, Smith K, Murphy PS, Ward E, Thibadeau J, et al. Weight Status of Children Participating in the National Spina Bifida Patient Registry. Pediatrics. 2022;150(6):e2022057007.

45. Boute T, Rizzo G, Mappa I, Makatsariya A, Toneto BR, Moron AF, et al. Correlation between estimated fetal weight and weight at birth in infants with gastroschisis and omphalocele. The Journal of Maternal-Fetal & Neonatal Medicine. 2022;35(16):3070-5.

46. Coenen H, Braun J, Köster H, Möllers M, Schmitz R, Steinhard J, et al. Role of umbilicocerebral and cerebroplacental ratios in prediction of perinatal outcome in FGR pregnancies. Archives of Gynecology and Obstetrics. 2022:1-10.

47. Ozler S, Kozanhan B, Bardak O, Arıkan MN. Are body roundness index and a body shape index in the first trimester related to foetal macrosomia? Journal of Obstetrics and Gynaecology. 2022;42(3):396-402.

48. Roeckner JT, Odibo L, Odibo AO. The value of fetal growth biometry velocities to predict large for gestational age (LGA) infants. The Journal of Maternal-Fetal & Neonatal Medicine. 2022;35(11):2099-104.

49. Visentin S, Londero AP, Cataneo I, Bellussi F, Salsi G, Pilu G, et al. A prenatal standard for fetal weight improves the prenatal diagnosis of small for gestational age fetuses in pregnancies at increased risk. BMC Pregnancy and Childbirth. 2022;22(1):1-7.

50. Song J, Liu J, Liu L, Jiang Y, Zheng H, Ke H, et al. The birth weight of macrosomia influence the accuracy of ultrasound estimation of fetal weight at term. Journal of Clinical Ultrasound. 2022;50(7):967-73.

51. Ng HM, MacDonell S, Yap J, Peddie MC, Scott T, Haszard JJ. Predicting height from ulna length for the determination of weight status in New Zealand adolescents: A cross‐sectional study. Journal of Human Nutrition and Dietetics. 2022;35(2):406-14.

52. Wahab RJ, Jaddoe VW, Voerman E, Ruijter GJ, Felix JF, Marchioro L, et al. Maternal body mass index, early-pregnancy metabolite profile, and birthweight. The Journal of Clinical Endocrinology & Metabolism. 2022;107(1):e315-e27.

53. Zhang Y, Wang Q, Xue M, Pang B, Yang M, Zhang Z, et al. Identifying factors associated with central obesity in school students using artificial intelligence techniques. Frontiers in Pediatrics. 2022;10:1060270.

54. Byeon H. Predicting South Korean adolescents vulnerable to obesity after the COVID-19 pandemic using categorical boosting and shapley additive explanation values: A population-based cross-sectional survey. Frontiers in Pediatrics. 2022;10:955339.

55. Carrillo-Balam G, Doi L, Marryat L, Williams AJ, Bradshaw P, Frank J. Validity of Scottish predictors of child obesity (age 12) for risk screening in mid-childhood: a secondary analysis of prospective cohort study data—with sensitivity analyses for settings without various routinely collected predictor variables. International Journal of Obesity. 2022;46(9):1624-32.

56. Wang Q, Yang M, Pang B, Xue M, Zhang Y, Zhang Z, et al. Predicting risk of overweight or obesity in Chinese preschool-aged children using artificial intelligence techniques. Endocrine. 2022;77(1):63-72.

57. Gomes D, Le L, Perschbacher S, Haas NA, Netz H, Hasbargen U, et al. Predicting the earliest deviation in weight gain in the course towards manifest overweight in offspring exposed to obesity in pregnancy: a longitudinal cohort study. BMC medicine. 2022;20(1):1-18.

58. He X, Jiang Z, Wu C, Zeng L, Qi M, Sun Y, et al. Development of a nutritional risk screening tool for preterm children in outpatient settings during a complementary feeding period: a pilot study. BMC pediatrics. 2022;22(1):1-12.

59. Huang K-H, Chen F-Y, Liu Z-Z, Luo J-Y, Xu R-L, Jiang L-L, et al. Prediction of pre-eclampsia complicated by fetal growth restriction and its perinatal outcome based on an artificial neural network model. Frontiers in Physiology. 2022:2272.

60. Lugo-Martinez J, Xu S, Levesque J, Gallagher D, Parker LA, Neu J, et al. Integrating longitudinal clinical and microbiome data to predict growth faltering in preterm infants. Journal of Biomedical Informatics. 2022;128:104031.

61. Price CR, Roeckner J, Odibo L, Odibo A. Comparing fetal biometric growth velocity versus estimated fetal weight for prediction of neonatal small for gestational age. The Journal of Maternal-Fetal & Neonatal Medicine. 2022;35(20):3931-6.

62. Han JH, Yoon SJ, Lee HS, Park G, Lim J, Shin JE, et al. Application of Machine Learning Approaches to Predict Postnatal Growth Failure in Very Low Birth Weight Infants. Yonsei Medical Journal. 2022;63(7):640.

63. Wang D, Schwinger C, Urassa W, Berhane Y, Strand TA, Fawzi WW. Comparing Attained Weight and Weight Velocity during the First 6 Months in Predicting Child Undernutrition and Mortality. The Journal of Nutrition. 2022;152(1):319-30.

64. Pradhan A, Mishra P, Tiwari S, Choure K, Gupta A. Prediction of Low Birth Weight by Quadruple Parameters in High-Risk Pregnancies. International Journal of Applied and Basic Medical Research. 2022;12(4):277.

65. Perichart-Perera O, Avila-Sosa V, Solis-Paredes JM, Montoya-Estrada A, Reyes-Muñoz E, Rodríguez-Cano AM, et al. Vitamin D Deficiency, Excessive Gestational Weight Gain, and Oxidative Stress Predict Small for Gestational Age Newborns Using an Artificial Neural Network Model. Antioxidants (Basel). 2022;11(3).

66. Chen Y, Cai C, Tan J, Lei X, Chen Q, Zhang J, et al. High-risk growth trajectory related to childhood overweight/obesity and its predictive model at birth. The Journal of Clinical Endocrinology & Metabolism. 2022;107(10):e4015-e26.
